# Genetic Ablation of the MET Oncogene Defines a Crucial Role of the HGF/MET Axis in Cell-Autonomous Functions Driving Tumor Dissemination

**DOI:** 10.3390/cancers15102742

**Published:** 2023-05-13

**Authors:** Chiara Modica, Marco Cortese, Francesca Bersani, Andrea Maria Lombardi, Francesca Napoli, Luisella Righi, Riccardo Taulli, Cristina Basilico, Elisa Vigna

**Affiliations:** 1 Candiolo Cancer Institute, FPO-IRCCS, 10060 Candiolo, TO, Italy; modicachiara89@gmail.com (C.M.); marco.cortese@ircc.it (M.C.); cribasilico@gmail.com (C.B.); 2 Department of Oncology, University of Torino, 10043 Orbassano, TO, Italy; francesca.bersani@unito.it (F.B.); andreamaria.lombardi@unito.it (A.M.L.); francesca.napoli@unito.it (F.N.); luisella.righi@unito.it (L.R.); riccardo.taulli@unito.it (R.T.)

**Keywords:** metastasis, invasion, MET, HGF, pancreatic cancers

## Abstract

**Simple Summary:**

The MET receptor and its ligand Hepatocyte Growth Factor (HGF) sustain cell proliferation, survival, motility, and invasion. Several pieces of evidence suggest that these biological activities support the metastatic potential of transformed cells. Cancer cell dissemination is sustained by cell-autonomous (executed by cancer cells) and non-cell-autonomous (executed by cells composing the tumor microenvironment) functions. To define the role of the HGF/MET axis in this complex process, we genetically knocked out the *MET* gene in cancer cells that were not dependent on MET signaling for their growth. In this way, we exclusively evaluated the relevance of the HGF/MET axis in prompting cell-autonomous activities (MET signaling in tumor microenvironment cells is maintained intact) independently of their intrinsic ability to proliferate (that is sustained by different oncogenic drivers). Our results proved that HGF/MET axis plays a crucial role in directing cell-autonomous functions regulating cancer cell dissemination, supporting a possible use of MET inhibitors in metastatic cancers.

**Abstract:**

Cancer cell dissemination is sustained by cell-autonomous and non-cell-autonomous functions. To disentangle the role of HGF (Hepatocyte Growth Factor) and MET ligand/receptor axis in this complex process, we genetically knocked out the MET gene in cancer cells in which MET is not the oncogenic driver. In this way, we evaluated the contribution of the HGF/MET axis to cancer cell dissemination independently of its direct activities in cells of the tumor microenvironment. The lack of MET expression in MET^−/−^ cells has been proved by molecular characterization. From a functional point of view, HGF stimulation of MET^−/−^ cancer cells was ineffective in eliciting intracellular signaling and in sustaining biological functions predictive of malignancy in vitro (i.e., anchorage-independent growth, invasion, and survival in the absence of matrix adhesion). Cancer cell dissemination was assessed in vivo, evaluating: (i) the ability of MET^−/−^ lung carcinoma cells to colonize the lungs following intravenous injection and (ii) the spontaneous dissemination to distant organs of MET^−/−^ pancreatic carcinoma cells upon orthotopic injection. In both experimental models, MET ablation affects the time of onset, the number, and the size of metastatic lesions. These results define a crucial contribution of the HGF/MET axis to cell-autonomous functions driving the metastatic process.

## 1. Introduction

The MET oncogene encodes for a transmembrane tyrosine kinase, the hepatocyte growth factor (HGF) receptor. HGF binding to MET leads to receptor dimerization and phosphorylation of major and docking sites, thereby activating downstream signaling pathways [1]. The HGF/MET axis elicits a complex program known as ‘invasive growth’ that produces a multifaceted biological outcome [2]. During ‘invasive growth’, activation of the proliferative response is coupled to cell survival, dissociation, scattering, extracellular matrix degradation, induction of cell polarity, migration, and dissemination at distant sites. All these processes, which are necessary and useful during embryonal development, tissue homeostasis, and wound healing, are aberrantly harnessed by cancer cells during tumor progression. In a limited number of cases, accounting for 2–3% of total cancers (COSMIC database: www.sanger.ac.uk, accessed on 10 September 2021), MET is the driver gene of malignancy, and this status (MET addiction) is strictly related to the presence of MET gene lesions [3]. Nevertheless, MET activation is not only relevant in cases of oncogene addiction: indeed, cancer cells exploit the invasive growth program to arrange and sustain an adaptive response to adverse microenvironmental conditions. Through MET signaling, the tumor survives under hypoxic conditions [4], fights immune attacks [5,6], resists radiotherapy and chemotherapy [7,8], and sustains the clonal expansion occurring after resistance to targeted treatments [9,10,11]. Thus, when a cancer cell needs to escape stress barriers, MET can confer extremely relevant properties to maintain the malignant phenotype and, as a consequence, disease progression. In this situation, MET is not a driver but an extremely useful expedient (‘MET expedience’) exploited by transformed cells [12]. ‘MET expedience’ activity has been considered pro-metastatic in many tumor types—including colon–rectal, gastric, ovarian, and pancreatic cancers—and it correlates with a more aggressive phenotype and worse clinical prognosis [13,14,15]. We previously demonstrated that concomitant targeting of HGF and MET allows optimal blockage of the ligand/receptor axis, strongly reducing the metastatic spreading of pancreatic cancer cells [16,17]. These results were obtained by a pharmacological approach, combining the activity of a Fab fragment derived from a MET inhibitory antibody, MvDN30 [18], with a mutated soluble MET ectodomain, decoyMET^K842E^ [16]. In line with these findings, Xu et al. showed that blockade of the HGF/MET axis inhibition, achieved by the concomitant use of a MET tyrosine kinase inhibitor and of an HGF-blocking antibody, in combination with a chemotherapeutic treatment [19], resulted in the abolition of pancreatic cancer metastasis. These strategies block both cell-autonomous and non-cell-autonomous MET functions. In the present study, we aimed to dissect the metastatic process by exclusively evaluating the contribution of MET signaling to cell-autonomous functions without interferences from MET activation occurring in the microenvironment. This was achieved by blocking the ligand/receptor axis through a genetic approach. Using CRISPR-Cas9 technology, we generated MET knock-out cancer cell lines and evaluated their invasive properties in vitro and their dissemination ability in vivo. We employed cancer cell lines in which MET is not the oncogenic driver. They carry a wild-type MET receptor, which is not constitutively phosphorylated, and it is converted to the active state upon paracrine ligand stimulation because the two selected cell lines do not express HGF (see https://sites.broadinstitute.org/ccle/, accessed on 8 February 2023). As it is known that the human MET receptor is not fully activated by murine HGF [20,21,22], animal experimentation was performed using genetically modified immunocompromised mice expressing human HGF in place of the murine ligand (hHGF-KI SCID mice) [23]. Thus, proper paracrine MET activation takes place in the human cancer cells transplanted into these animals [16,17], allowing us to fully estimate the contribution of the HGF-MET axis to the cell-autonomous functions involved in the metastatic process.

## 2. Materials and Methods

### 2.1. Cell Culture

A549 human lung adenocarcinoma cells and Capan-I human pancreatic adenocarcinoma cells were obtained from ATCC/LGC Standards S.r.l. (Sesto San Giovanni, Italy), and cultured as suggested by the supplier. All cell cultures were routinely tested for mycoplasma contamination.

### 2.2. Generation and Genetic Validation of MET Knock-Out in Cancer Cell Clones

A number of gRNAs targeting human MET were designed using the Guide Design Resource online tool (https://zlab.bio/guide-design-resources, accessed on 11 December 2018). The following gRNA sequence was used: 5′-GGTGTTTCCGCGGTGAAGTT-3′. Oligos were annealed and cloned into the pSpCas9(BB)-2A-Puro (PX459) plasmid (ALL-IN-ONE vector strategy, Addgene; 62988) into the BbsI restriction site using a standard protocol [24]. A549 or Capan-I cells were transfected with the generated plasmid using TransIT-X2 Transfection Reagent (Mirus Bio, Madison, WI, USA), according to the protocol of the supplier. After 48 h, a fresh complete cell culture medium with Puromycin (1 μg/μL; Sigma-Aldrich, St. Luois, MO, USA) was added to the cells for 10 days. Puromycin-resistant cells underwent limiting dilution to obtain a monoclonal cell population. DNA was extracted from the single cell clones, negative for MET expression, using Maxwell RSC Tissue DNA kit (Promega Corp., Madison, WI, USA)); the region of interest was amplified by PCR using the following primers: forward 5’-CTTGCACCTGGCATCCTC-3’; reverse 5′-CAGTCCTGACATGGGAAACA-3’. Then, the PCR product was cloned into the TOPO vector (Topo TA Cloning KIT, Thermo Fisher Scientific Inc., Monza, Italy) according to the manufacturer’s protocol. Next, 70 single colonies were picked, and the plasmids re-isolated by minipreps (Quick Plasmid Miniprep Kit-Invitrogen Corp., Waltham, MA, USA) were then individually sequenced using the primers reported above to confirm the genetic lesion on the MET gene by Sanger sequencing.

### 2.3. ELISA Assay

A 96-well ELISA plate was coated with 100 ng/mL of an antibody that recognizes the extracellular domain of MET (AF276—R&D Systems, Minneapolis, MN, USA). After saturation with 0.5% BSA in PBS, cell lysates obtained from each clone with the InstanOne ELISA Cell Lysis Buffer (Invitrogen Corp.) were transferred to the plate. The presence of MET receptor in the cell lysate was revealed by the anti-MET antibody DO24 (100 ng/well, [25]) followed by an HRP-conjugated anti-mouse antibody (GE Healthcare Italia, Milano, Italy). After the addition of the TMB substrate (Sigma-Aldrich), the signal from the colorimetric reaction was quantified by the multilabel reader VICTOR X4 (PerkinElmer Inc., Waltham, MA, USA). As a reference, a sample lysate from the respective wild-type cell line was included in the assay.

### 2.4. Flow Cytometry Analysis

Cells were detached with PBS/EDTA 1% and stained with APC- or PE-conjugated mouse anti-Human MET (3D6; BD Biosciences Italia, Milano, Italy). For isotype control, an APC- or PE-conjugated anti-mouse Ig antibody (BD Biosciences) was used. Cells were co-stained with DAPI (Sigma-Aldrich). Labelled cells were acquired on a CyAn ADP flow cytometer (Beckman Coulter Italia, Cassina de’ Pecchi, Italy), and MET expression was analyzed by Summit v4.3 software (Dako-Beckman Coulter Italia). The signal derived from the isotype control was set as 0  <  MFI  <  10^1^, and cells were considered MET positive when MFI  >  10^1^.

### 2.5. Western Blot Analysis

For analysis of HGF-dependent MET activation, sub-confluent cell monolayers were maintained in the absence of serum for 24 h, then stimulated with HGF (100 ng/mL—R&D Systems) for 15 min at 37 °C. Cell lysates were obtained using Laemmli buffer. Equal amounts of total proteins (20 µg/sample) were resolved by SDS-PAGE and transferred to 0.2 µm nitrocellulose Trans-Blot Turbo TM membranes (Thermo Fisher Scientific Inc.). For protein detection, the following antibodies were used: anti-MET phospho-Tyr1234/1235 (D26); anti-MET (D1C2); anti-pAKT (Ser473), anti-AKT, anti-pERK1/2 (Thr202/Tyr204), anti-ERK1/2 polyclonal Abs (all from Cell Signaling Technology, Beverly, MA, USA); anti-vinculin (clone hVIN-1) and anti-alpha actin (both from Sigma Life Science, St. Luois, MO, USA). All antibodies were applied according to the protocols supplied by the manufacturers. After incubation with appropriate HRP-conjugated secondary antibodies (all from GE Healthcare) and the ECL reagent (Promega Corp.), Western blot bands were detected by ChemiDoc Touch Imaging System (Bio-Rad Laboratories, Segrate, Italy).

### 2.6. Cell Growth Assay

Cells were seeded in a 96-well plate (A549: 2000 cells/well; Capan-I: 4000 cells/well) in a complete culture medium without or with HGF (25 ng/mL). Cell growth was evaluated after fixed time points (24, 48, and 96 h) by CellTiter-Glo luminescent cell viability assay (Promega Corp.), according to the manufacturer’s instructions. Chemiluminescence was detected with VICTOR X4.

### 2.7. Cell Proliferation Assay

Cells (10^5^/well) were seeded in a six-well plate in a complete culture medium. After 24 h, cells were washed with PBS, and the medium was replaced with fresh serum-free medium. After 24 h, HGF (25 ng/mL) was added to the culture (medium without FCS in the case of A549 and medium with 2% FCS in the case of Capan-I). Cell proliferation was evaluated by measuring DNA synthesis by EdU incorporation using the Click-iT^®^ EdU Alexa Fluor^®^ 647 Flow Cytometry Assay Kit (Thermo Fisher Scientific Inc.), according to the manufacturer’s instruction. Briefly, after 48 h of culture, 10 µM EdU was added to the culture medium. After 1 h, cell monolayers were washed and detached by incubation with Accutase (Sigma-Aldrich). Collected cells were fixed with Click-iT^®^ fixative solution and then permeabilized by 15 min incubation with Click-iT^®^ saponin-based permeabilization buffer. After washing with PBS, cells were incubated for 30 min with the Click-iT^®^ reaction cocktail. Labeled cells were acquired on a Cyan ADP flow cytometer and analyzed by Summit v4.3 software.

### 2.8. Cell Cycle Analysis

Cells were seeded in six-well plates in a complete culture medium. After 24 h, cells were washed with PBS, and the medium was replaced with fresh serum-free medium. After 24 h, cell monolayers were washed, and a 2% FCS medium with or without HGF (25 ng/mL) was added to the culture. After 48 h, monolayers were trypsinized, harvested, and washed with PBS. Equal amounts of cells for each condition (5 × 10^5^) were stained with propidium iodide (50 μg/ml; Merck Life Science Italia, Milano, Italy) in the presence of RNase A (0.25 mg/mL; Merck Life Science) in citrate buffer. After a 15 min incubation at room temperature in the dark, the cell cycle distribution was assessed by flow cytometry with FACSDiva v9.2 softwareon a BD FACSCelesta Cell Analyzer (BD Biosciences). Post-acquisition analysis was performed using FlowJo v10.8.1 software (BD Biosciences).

### 2.9. Clonogenic Assay

MET wild-type and MET^−/−^ A549 cells were seeded in 4 or 20 cells/well in a 24-well plate in a complete culture medium. After 10 days of culture, cells were fixed with 11% glutaraldehyde (Sigma-Aldrich) and stained with 0.1% crystal violet (Sigma-Aldrich). The number of clones grown in each well was scored by observation using a light microscope (Leica Biosystems, Buccinasco, Italy). Plating efficiency was determined by dividing the average number of clones by the original number of seeded cells.

### 2.10. Anchorage-Independent Cell Growth Assay

For anchorage-independent growth assays, cells were seeded in 48-well dishes (1000 cells/well) in a culture medium containing 2% FBS and 0.5% SeaPlaque agarose -BMA (Sigma-Aldrich), without or with HGF (25 ng/mL). Medium has been changed twice a week. After 21 days of culture, colonies were stained by tetrazolium salts (Sigma-Aldrich) and scored with LIPSI High Content Screening Microscope (Nikon Healthcare, Amstelveen, The Netherlands).

### 2.11. Generation of MET^−/−^ Cells Re-Expressing the MET Receptor

MET^−/−^ A549 cancer cells were transduced with 100 ng/mL p24 of lentiviral vectors carrying the MET gene under the control of a tetracycline (TET)-inducible promoter (TET-off system), as described in [26]. MET receptors exposed at the cell surface were evaluated by cytofluorimeter analysis as described above. A total of 10^7^ cells stained with APC-conjugated anti-MET antibodies were then resuspended in PBS containing 4mM EDTA, 1% FCS and 0.01% StemPro Accutase (Thermo Fischer Scientific Inc.) and sorted according to high MET expression using the BD FACS Melody cell sorter (BD Biosciences). MET expression on pre-sorting and sorted cell populations were analyzed using FlowJo v10.8.1 software.

### 2.12. Invasion Assay

A549 (7 × 10^4^/well) or Capan-I (1 × 10^5^/well) cells, either wild-type or MET^−/−^, were resuspended in cell culture medium with 1% FBS and seeded in the upper compartment of transwell chambers (8.0 μm pore polycarbonate membrane insert; Corning Inc., New York, NY, USA) pre-coated with 40 μg/well of Matrigel Reduced Growth Factors (Corning Inc.). The lower compartment of the chamber was filled with 1% FBS cell culture medium supplemented with HGF (25 ng/mL). For A549, either wild-type, MET^−/−^ or MET^−/−^ back-expressing MET upon lentiviral vector transduction, the following conditions have also been tested: lower chamber medium + 5% FCS + HGF (25 ng/mL); upper chamber medium + 1% FCS + HGF (25 ng/mL). After 24 h, cells on the upper side of the transwell filters were mechanically removed, while cells migrated through the membrane were fixed with 11% glutaraldehyde and stained with 0.1% crystal violet. All images were captured with an optical microscope (Leica Biosystems). Images were quantified with Image-J v1.59s software.

### 2.13. Anoikis Assay

Wild-type or MET^−/−^ A549 (5 × 10^4^ cells/well) or Capan-I (10^5^ cells/well) cells were seeded in six-well ultralow attachment surface plates in medium plus 2% FCS (A549) or 20% FCS (Capan-I), with or without HGF (25 ng/mL). After 24 h (A549) or 48 h (Capan-I), cell suspensions were collected, washed in PBS, and resuspended in 100 μL of Annexin V binding buffer (BD Biosciences) containing 2.5 μL of biotin-conjugated Annexin V (BD Biosciences) and incubated for 15 min at room temperature. Following one wash in PBS, cells were incubated with 0.5 μL of streptavidin–allophycocyanin (APC; BD Biosciences) in 100 μL of Annexin V binding buffer (BD Biosciences) for an additional 15 min at room temperature in the dark. Then, 7-AAD dye (BD Biosciences) was added right before the acquisition with FACSDiva v9.2 software on a BD FACSCelesta Cell Analyzer. Post-acquisition analysis was performed using FlowJo v10.8.1 software.

### 2.14. Generation of Luciferase-Expressing Cells

For expression of the luciferase gene, wild-type or MET^−/−^ A549 or Capan-I cells were transduced with 100 ng/mL p24 of lentiviral vectors carrying the luciferase gene under the control of the CMV promoter [27] as described [28]. Luciferase expression was evaluated in vitro on different amounts of cells by adding XenoLight D-Luciferin (150 ng/mL; PerkinElmer Inc.) to the culture medium. The bioluminescent signal was measured with VICTOR X4 multilabel plate reader (PerkinElmer Inc.).

### 2.15. In Vivo Experiments

All in vivo experiments were performed according to protocols approved by the Ethical Committee for animal experimentation of the Fondazione Piemontese per la Ricerca sul Cancro and by the Italian Ministry of Health. The hHGF-KI SCID mice were obtained from AVEO Pharmaceuticals, Cambridge, MA, USA. For the lung colonization experiment, 2 × 10^6^ cells resuspended in 200 µL of PBS were injected into the tail vein of four-to-six-week-old male hHGF-KI SCID mice. XenoLight D-Luciferin (150 mg/kg) was injected intraperitoneally in mice 4 h, 4, 8, and 16 days after cells injection, and the bioluminescent signal was measured by IVIS SpectrumCT in vivo imaging system (PerkinElmer Inc.) on live animals. Five weeks after cell injection, mice were injected with XenoLight D-Luciferin and euthanized, and the bioluminescence signal was measured in the isolated lungs. All IVIS data were analyzed by Living Image v4.5 software (PerkinElmer Inc.). Lungs were also fixed and paraffin-included for histological and immunohistochemical analyses.

For the pancreas orthotopic experimental model, luciferase-expressing Capan1 cells (10^5^ cells/mouse in 25 µL of serum free-medium + 25 µL Matrigel matrix) were injected in the pancreas of four-to-six-week-old female hHGF-KI SCID mice. After three days, XenoLight D-Luciferin was injected intraperitoneally in mice. At sacrifice, five weeks after cell injection, XenoLight D-Luciferin was administered to mice, and the bioluminescent signals of tumors and isolated organs were measured by IVIS SpectrumCT. All IVIS data were analyzed by Living Image v4.5 software. Collected pancreases were also fixed, and paraffin-included for histological analysis.

### 2.16. Immunohistochemistry Staining

Four μm-thick sections obtained from paraffin-embedded lungs collected from mice injected with A549 cells and charged on slides were subjected to immunohistochemical analyses using an automated platform (OMNIS, Dako-Beckman Coulter Italia). Ki67 positivity was detected with a specific FLEX Monoclonal Mouse Anti-Human Ki-67 Antigen, Clone MIB-1, Ready-to-Use kit (Dako-Beckman Coulter Italia). Quantification of Ki67 staining was completed by ImageJ v1.59s software by calculating the percentage of positive nuclei with respect to the number of total nuclei within ten malignant nodules from different lung samples. MET protein was detected by recombinant anti-Met antibody clone SP44 (1:200 dilution; Abcam, Cambridge, UK). For phospho-MET detection, the antigen was unmasked, incubating the slides in citrate buffer pH 6.6 at 98 °C for 1 h and then stained with anti-human/mouse phospho-MET (Tyr 1234–1235) polyclonal antibodies AF2480 (1:100 dilution, O/N at 4 °C; R&D Systems) followed by anti-rabbit-HRP conjugated antibodies (GE Healthcare).

### 2.17. Statistical Analysis

Average, Standard Deviation (SD), and Standard Error of the Mean (SEM) were calculated using Microsoft Office Excel 2010 software (Microsoft Corp., Redomond, WA, USA) or GraphPad Prism v.9 software (GraphPad Software, Boston, MA, USA). Statistical significance was determined using GraphPad Prism v.9 software by the two-tailed Student’s *t*-test or Mann–Whitney test. For cell cycle analyses, the significance has been evaluated by comparing the G0/G1 phase values and the sum of S + M/G2 phase values. A value of *p* ≤ 0.05 was considered significant.

## 3. Results

### 3.1. Generation and Validation of a MET Knock-Out Human Lung Carcinoma Cell Line

**Figure 1:**
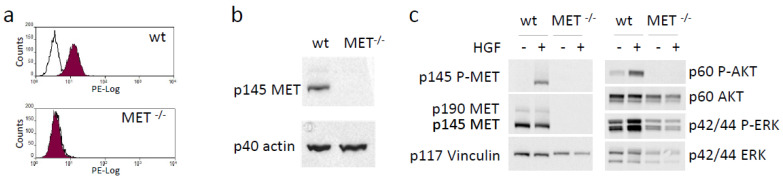
Generation and validation of a MET^−/−^ NSCLC cell line. (**a**) Flow cytometry analysis of MET expression at the surface of wild-type (wt) and MET^−/−^ A549 cells. (**b**) Immunoblotting analysis of MET expression in lysates from wild-type (wt) and MET^−/−^ A549 cells. Actin was used as a loading control. (**c**) HGF-dependent phosphorylation of MET and activation of downstream transducers in lysates from wild-type (wt) and MET^−/−^ A549 cells. Vinculin was used as a loading control. p145 MET: MET receptor β chain; p145 P-MET: phosphorylated MET receptor β chain; p190 MET: precursor form of the MET receptor; p60AKT: AKT; p60 P-AKT: phosphorylated AKT; p44/42ERK: ERK; p44/42 P-ERK: phosphorylated ERK; p40 actin: Actin; p117 Vinculin: vinculin. Data reported in the figure are representative of at least two experiments. The uncropped Western blots are shown in Supplementary Materials File S1.

We functionally disabled *MET* alleles in A549 human lung cancer cells by exploiting CRISPR-directed gene editing. We designed two MET-specific guide RNAs (gRNA_MET-A and gRNA_MET-B) targeting the second exon of the oncogene (Figure S1A), a region included in all the mRNA isoforms encoding a functional MET receptor. After transfection of A549, a human lung carcinoma cell line, with the appropriate gRNA-plasmid (Figure S1B), followed by puromycin selection and limiting dilution cloning, we obtained several clones that were analyzed for MET expression by an ELISA assay. One putative MET^−/−^ A549 clone was identified in the gRNA_MET-A clone group, while no knock-out clone was generated by gRNA_MET-B transfection (Figure S1C). The ablation of the MET protein in MET^−/−^A549 was assessed by flow cytometry (Figure 1a) and immunoblotting analysis (Figure 1b). To rule out the presence of a form of MET generated by gene editing not undetectable by the antibodies used in the above-described assays but still competent to elicit intracellular signaling, we treated MET^−/−^ A549 cells with HGF and checked the activation of AKT and ERK, the main transducers downstream to MET. HGF stimulation of MET^−/−^ A549 cells did not activate any full-length MET receptor and did not modify the phosphorylation levels of the analyzed signaling transducers, confirming, also from a functional point of view, the complete knock-out of the HGF/MET axis (Figure 1c).

### 3.2. Genetic MET Ablation Impaired the HGF-Driven Malignant Phenotype of Lung Carcinoma Cells In Vitro

**Figure 2:**
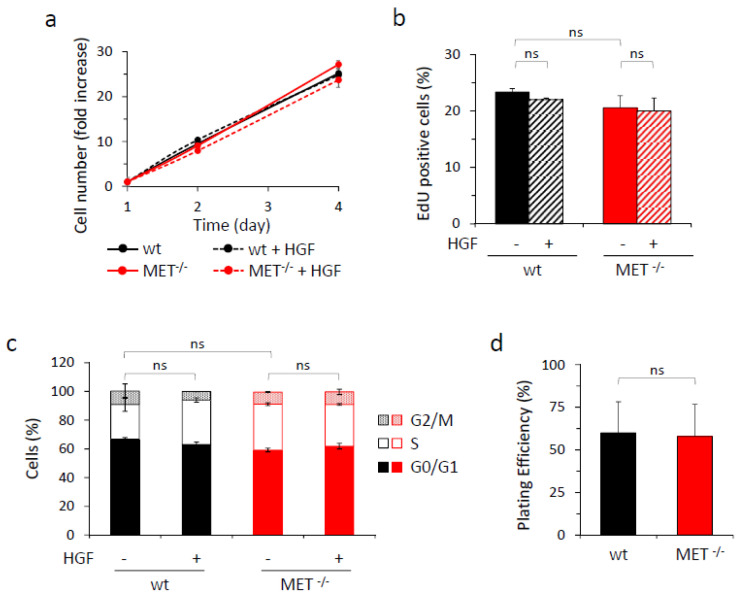
In vitro analysis of growth properties of wild-type and MET^−/−^ A549 cells. (**a**) Analysis of viability by quantitation of metabolically active wild-type (wt) or MET^−/−^ A549 cells treated or not with HGF (25 ng/mL) after two or four days of culture. Graph reports the fold increase in cell number with respect to day one. Each point is the mean of triplicate values; bars represent SD. (**b**) Analysis of cell proliferation by EdU incorporation in wild-type (wt) or MET^−/−^ A549 cells treated or not with HGF (25 ng/mL) after 48 h of culture in serum-free medium. Graph reports the percentage of EdU-positive cells. Each point is the mean of duplicate values; bars represent SD. (**c**) Cell cycle analysis of wild-type (wt) or MET^−/−^ A549 cells treated or not with HGF (25 ng/mL) after 48 h of culture in 2% serum medium. Graph reports the percentage of cells in each cell cycle phase. Each point is the mean of triplicate values; bars represent SD. (**d**) The clonogenic ability of wild-type (wt) or MET^−/−^ A549 cells after 10 days of culture in medium with 10% serum. Graph reports the percentage of plating efficiency. Each point is the mean of 10 values; bars represent SD. ns: not significant.

We then assessed the impact of MET gene knock-out on HGF-driven biological responses by performing different assays. Viability, proliferation rate—evaluated by cytofluorimeter analysis of EdU incorporation and cell cycle profile—and in vitro clonogenic capacity were comparable in MET-expressing and MET-deficient cells. Moreover, HGF treatment did not potentiate cell growth under standard culture conditions, nor in serum-free or low serum medium (Figure 2 and Figure S2).

**Figure 3:**
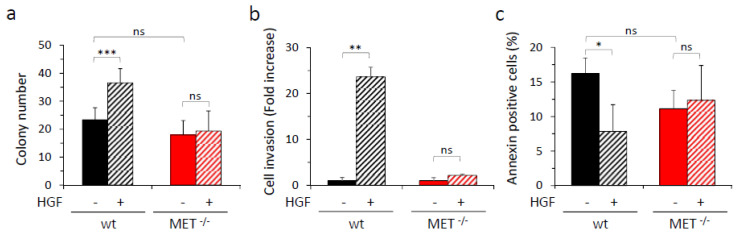
In vitro analysis of HGF-driven biological responses in wild-type and MET^−/−^ A549 cells. (**a**) Analysis of anchorage-independent growth by soft agar assay with wild-type (wt) or MET^−/−^ A549 cells. Graph reports the number of colonies obtained after 14 days of culture in the presence or in the absence of HGF (25 ng/mL). Each point is the mean of quadruplicate values. Each quadruplicate value is obtained by calculating the mean of the colony number present in at least three different fields of the same sample; bars represent SD. (**b**) Analysis of cell invasion by transwell assay with wild-type (wt) or MET^−/−^ A549 cells. Graph reports the fold increase in cells migrated through a Matrigel layer during 24 h of culture in the presence of HGF (25 ng/mL), with respect to the untreated counterpart. Each point is the mean of triplicates. (**c**) Analysis of apoptosis induced by loss of cell/matrix interaction (Anoikis) in wild-type (wt) or MET^−/−^ A549 cells. Graph reports the percentage of annexin-positive cells (early and late stages of apoptosis, i.e., Annexin V positive/7AAD negative plus Annexin V positive/7AAD positive cells), treated or not with HGF (25 ng/mL) after 24 h of culture in the absence of matrix adhesion. Each point is the mean of triplicate values; bars represent SD. ***, *p* ≤ 0.001; **, *p* ≤ 0.01; *, *p* ≤ 0.05; ns: not significant. Data reported in the figure are representative of at least two experiments.

Anchorage-independent growth, low in basal condition and comparable for the two analyzed cell populations, was pushed by HGF in the case of wild-type A549, while MET^−/−^ cells did not respond to the ligand (Figure 3a and Figure S3A). The analysis of migratory and invasive potential, evaluated by performing transwell assays with Matrigel-coated filters, showed that no significant differences between A549 wild-type and MET^−/−^ were scored under basal condition, while HGF treatment strongly stimulated wild-type cells but not MET^−/−^ cells, confirming that MET^−/−^ cells were completely unresponsive to the ligand (Figure 3b and Figure S3B). As a control, we rescued MET expression in A549 MET^−/−^ cells by transduction with MET-lentiviral vectors. MET re-expressing cells recovered HGF-induced invasive properties (Figure S4). We also tested the ability of wild-type and MET^−/−^ A549 cells to survive in the absence of matrix adhesion. While in wild-type A549 cells HGF was able to counteract apoptosis, MET^−/−^ cells did not benefit from the treatment (Figure 3c and Figure S5).

### 3.3. Genetic MET Ablation Strongly Reduced Lung Colonization by NSCLC Cells In Vivo

**Figure 4:**
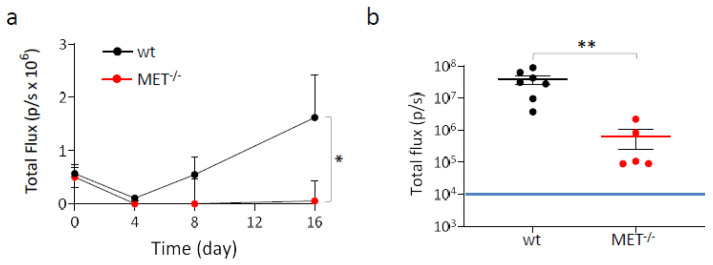
Lung colonization assay with MET^−/−^ A549 cells. Luciferase-expressing wild-type and MET^−/−^ A549 cells were injected into the tail vein of hHGF-KI mice. (**a**) IVIS analysis of mice performed 4 h post-injection (day zero) and then after 4-8-16 days. Each time point represents the mean value of the group. Bars represent SEM. (**b**) IVIS analysis of lungs excised from mice at day 36. Each dot represents the value of the lungs excised from one mouse. Black and red lines: average value for each group. Bars represent SEM. The blue line indicates the threshold (10^4^) below which IVIS values are considered negative. *, *p* ≤ 0.05; **, *p* ≤ 0.01. The data reported in the figure are representative of two experiments.

The metastatic potential of MET^−/−^A549 cells was assessed in vivo by measuring their ability to generate tumor nodules in the lungs after intravenous injection in hHGF-KI mice. These immunocompromised transgenic animals express physiological amounts of human HGF in place of the murine factor, a condition that overcomes the species-specificity issue of HGF/MET interaction [21,29]. This experimental model allows to correctly evaluate the contribution of the HGF/MET axis activation to the dissemination of human cancer cells. To enable detection by IVIS spectrum in vivo imaging system (IVIS), wild-type and MET^−/−^ A549 cells were genetically modified to express comparable levels of luciferase by means of lentiviral vector technology (Figure S6). Four hours after cell injection, mice were analyzed by IVIS (day zero); wild-type and MET^−/−^ A549 cells originated superimposable signals, indicating that the animals of the two groups received comparable amounts of cells. Subsequent analysis on day four showed that the signal decreased in both groups, presumably due to the massive death of the injected cells. After eight days, detectable lung colonies were present in five out of seven of the control mice, indicating that surviving wild-type A549 cells were growing; in contrast, the animals injected with MET^−/−^ cells were all negative. The difference between the two groups further increased on day 16, when a barely-detectable lung signal was measured only in one animal injected with cells not expressing MET, while mice in the control group displayed an average level of bioluminescence 30 folds higher (Figure 4a and Figure S7A). At the end of the experiment (day 36), lung colonization by MET^−/−^A549 cells was extremely reduced, as assessed by measuring the bioluminescent signals in the excised lungs (Figure 4b). Histological analysis confirmed the presence of a large number of tumor nodules in the lungs of mice injected with wild-type cells, while nodules originated from the injection of MET^−/−^ A549 cells were lower in number and smaller in size. In both experimental groups, proliferating cells were highlighted by Ki67 staining. The percentages of proliferating cells within the malignant lesions were not significantly different (24.4 ± 3.1 and 15.4 ± 11.5, wild-type and MET^−/−^ A549, respectively; *p* > 0.05), indicating that the intrinsic proliferation rate of the MET wild-type and MET^−/−^ tumors were comparable. The immunohistochemical analysis also confirmed the expression of MET only in wild-type tumors and revealed a variable but well-detectable phospho-MET signal, indicating that paracrine HGF expressed by the mouse stroma is effective in activating the MET receptor (Figure S7B).

### 3.4. Generation, Validation, and Biological Characterization of a MET Knock-Out Human Pancreatic Cancer Cell Line

**Figure 5:**
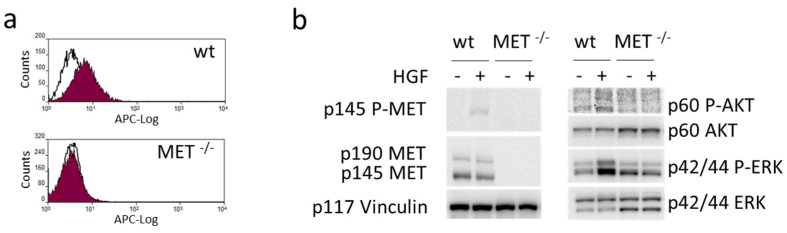
Generation and validation of a MET^−/−^ pancreatic carcinoma cell line. (**a**) Flow cytometry analysis of MET expression at the surface of wild-type (wt) and MET^−/−^ Capan-I cells. (**b**) HGF-dependent phosphorylation of MET and activation of downstream transducers in lysates from wild-type (wt) and MET^−/−^ Capan-I cells. Vinculin was used as a loading control. p145 MET: MET receptor β chain; p145 P-MET: phosphorylated MET receptor β chain; p190 MET: precursor form of the MET receptor; p60AKT: AKT; p60 P-AKT: phosphorylated AKT; p44/42ERK: ERK; p44/42 P-ERK: phosphorylated ERK; p117 Vinculin: vinculin. The uncropped Western blots are shown in Supplementary Materials File S1.

The plasmids expressing gRNA_MET-A and gRNA_MET-B were also transfected in Capan-I, a human PDAC cell line. After puromycin selection, the clones obtained by limiting dilution were analyzed for MET expression as described above. We obtained four putative MET^−/−^ Capan-I clones (Figure S8A). Further analysis by immunoblotting defined that only one clone was completely negative (i.e., clone 13, obtained with gRNA_MET-B) because a band with a molecular weight compatible with the MET precursor was detected in the others (Figure S8B). The absence of the MET receptor exposed at the cell surface was assessed by flow cytometry (Figure 5a). The immunoblotting analysis confirmed that no full-length MET receptor was expressed, and HGF-dependent activation of MET and its intracellular signaling did not take place in the selected MET^−/−^ Capan-I clone (Figure 5b).

**Figure 6:**
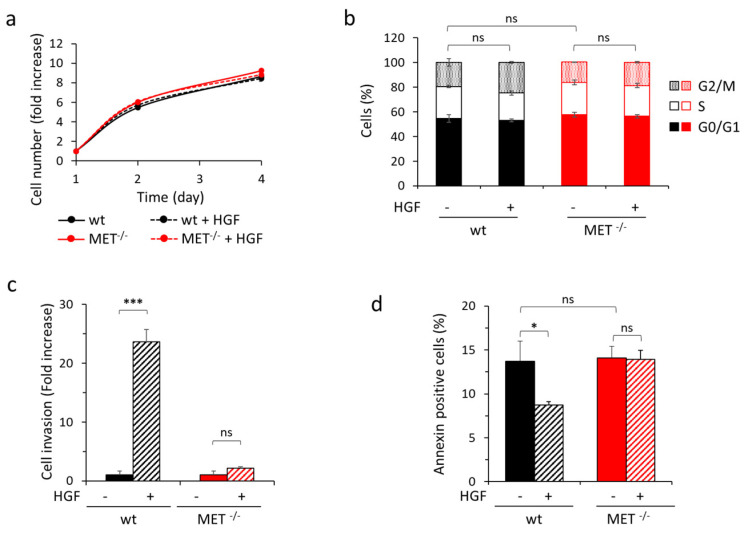
In vitro analysis of HGF-driven biological responses in wild-type and MET^−/−^ Capan-I cells. (**a**) Analysis of viability by quantitation of metabolically active wild-type (wt) or MET^−/−^ Capan-I cells treated or not with HGF (25 ng/mL) after two or four days of culture. Graph reports the fold increase in cell number with respect to day one. Each point is the mean of triplicate values; bars represent SD. (**b**) Cell cycle analysis of wild-type (wt) or MET^−/−^ Capan-I cells treated or not with HGF (25 ng/mL) after 48 h of culture in 2% serum medium. Graph reports the percentage of cells in each cell cycle phase. Each point is the mean of triplicate values; bars represent SD. (**c**) Analysis of cell invasion by transwell assay with wild-type (wt) or MET^−/−^ Capan-I cells. Graph reports fold increase in cells migrated through a matrigel layer during 24 h of culture in the presence of HGF (25 ng/mL), with respect to the untreated counterpart. Each point is the mean of duplicates. (**d**) Analysis of apoptosis induced by loss of cell/matrix interaction (Anoikis) in wild-type (wt) or MET^−/−^ Capan-I cells. Graph reports the percentage of annexin-positive cells (early and late stages of apoptosis, i.e., Annexin V positive/7AAD negative plus Annexin V positive/7AAD positive cells) after 48 h of culture in the absence of matrix adhesion, treated or not with HGF (25 ng/mL). Each point is the mean of triplicate values; bars represent SD. ***, *p* ≤ 0.001; *, *p* ≤ 0.05; ns: not significant. Data reported in the figure are representative of at least two experiments.

We then assessed the impact of MET gene knock-out on biological responses. The genetic modification did not influence the growth ability of MET^−/−^ Capan-I cells, as assessed by evaluation of metabolically active cells under standard culture conditions, nor proliferation, as assessed by EdU incorporation and by cell cycle analysis under low serum condition, either in the absence or in the presence of HGF (Figure 6a,b and Figure S9). On the contrary, stimulation of cell invasion and protection from anoikis by HGF were abolished (Figure 6c,d and Figure S10).

### 3.5. Genetic MET Ablation Strongly Reduced PDAC Cell Growth at the Orthotopic Site and Dissemination at Distant Organs In Vivo

**Figure 7:**
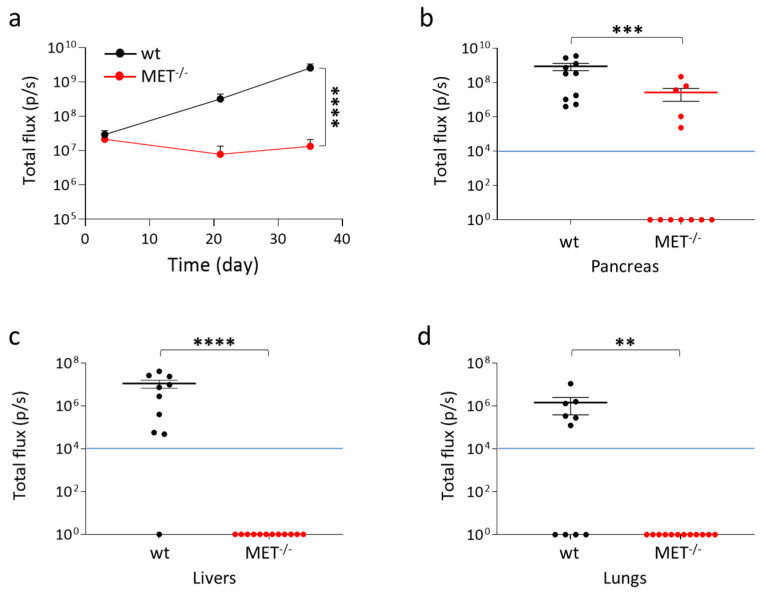
In vivo analysis of MET^−/−^ Capan-I tumors and metastasis. Luciferase-expressing wild-type and MET ^−/−^ Capan-I cells were injected into the pancreas of hHGF-KI mice. (**a**) IVIS analysis of mice performed 3, 21, and 35 days post-injection. Each time point represents the mean value of the group. Bars represent SEM. (**b**–**d**) IVIS analysis of isolated organs (pancreas, livers, and lungs) excised from mice at day 35. Each dot represents the value of the organ excised from one mouse. The blue lines indicate the threshold (10^4^) below which IVIS values are considered negative. ****, *p* ≤ 0.0001; ***, *p* ≤ 0.001; **, *p* ≤ 0.01. The data reported in the figure are representative of two experiments.

To assess the impact of MET knock-out in a model of spontaneous metastasis in vivo, wild-type and MET^−/−^ Capan-I cells were orthotopically injected in hHGF-KI mice. In order to allow direct measurement of tumor growth in vivo with the IVIS system, cells were engineered with lentiviral vectors to express luciferase at comparable levels (Figure S11). The animals were analyzed for luciferase signals at different time points after cell injection. While wild-type Capan-I cells grew in vivo, as demonstrated by the time-dependent increase of IVIS signal, MET^−/−^ Capan-I cells did not expand, as the IVIS signal remained stable over time (Figure 7a). After 35 days, mice were sacrificed, and luciferase was measured in the isolated pancreases. Seven out of twelve animals injected with MET^−/−^ Capan-I cells (58.3%) were tumor negative, and among the five tumor-positive mice, two masses were smaller in size than the smallest masses observed in the wild-type group (Figure 7b and Figure S12). Histological analysis of the samples confirmed that the majority of mice injected with MET^−/−^ Capan-I cells were tumor-free. The few lesions observed were localized, while in the case of wild-type tumors, cancer cells appeared to be infiltrated throughout the organ parenchyma, making it extremely difficult to highlight the presence of normal tissue. MET^−/−^ cells displayed impaired cell proliferation, as demonstrated by mitotic cell analysis (Figure S13). The highly aggressive wild-type tumors showed necrotic areas: on the contrary, these were completely absent in MET^−/−^ tumors. At sacrifice, we also checked the presence of metastatic nodules in excised lungs and livers, which represents the preferential sites of PDAC cell dissemination. The metastatic ability of MET^−/−^ Capan-I cells was deeply impaired as no IVIS-detectable lesions were measured (Figure 7c,d and Figure S12).

## 4. Discussion

In this study, we applied CRISPR-Cas9 mediated knocking-out of the *MET* gene in cancer cells to cast light on the role played by the HGF/MET axis as a key regulator of cell-autonomous metastatic properties. The currently available data defining the biological role of MET as a master driver of the invasive growth program strongly indicates that this receptor can be crucial during tumor progression, favoring the malignant phenotype. This notion has been sustained by the application of molecules with HGF/MET inhibitory properties in experimental in vivo models [16,17,19]. Nevertheless, pharmacological inhibition blocks MET activation in both cancer cells and cells populating the tumor microenvironment, while genetic manipulation resulting in MET knock-out in cancer cells narrows down the analysis to the tumor, allowing the dissection of cell-autonomous and non-cell-autonomous functions. Moreover, although gene ablation can be considered artificial, the complete absence of a functional protein eliminates issues intrinsic to the application of molecules with inhibitory activity related to their efficacy, specificity, and toxicity, guaranteeing an easier interpretation of the results. Thus, gene ablation represents a reliable tool to generate knowledge and proof of concept.

The well-known and highly accepted ‘seed and soil’ theory [30] is based on the concept that the success of the metastatic process is defined by features characterizing both the transformed cell (the seed) and the environment into which the cancer cell is landed (the soil). In this context, MET ablation in cancer cells allows the role of MET to be explored only in the context of ‘the seed’, removing both intrinsic and extrinsic seed features [31]. The first group includes peculiar traits of the transformed cell that determine its malignant level (i.e., EMT grade, stem features, persistency potential). The second group includes the aptitude of cancer cells to directly or indirectly induce the release of factors, such as endosomes, cytokines, chemokines, and other molecules, which act on the surrounding environment either at the primary site, favoring invasion and intravasation of the transformed cells, and/or at the distant site, educating the pre-metastatic niche to cancer cell accommodation. Relying on previous literature, MET has been linked to both intrinsic and extrinsic factors [32,33,34,35]. As the results reported here are not suitable to define if MET plays a prominent role in one of the two aspects, the investigation of this point will require future studies.

It has been reported that the new colonization site can be favorably conditioned by stromal cells originally present at the primary site and re-localized at the metastatic nodule by traveling associated with the cancer cells [36,37]. Thus, not-transformed cells clustered together with transformed cells remarkably facilitate metastatic seeding. In this context, MET could be one of the molecules directly or indirectly involved in maintaining structurally intact the tumor fragment shed from the primary lesion.

The results of the lung colonization experiments show that wild-type A549 cells are trapped in the lungs and rapidly graft in the pulmonary parenchyma, while the formation of lung nodules by MET^−/−^ A549 cells is dramatically impaired. Although the delivery of cancer cells via the vascular system does not fully recapitulate all the steps defining the process of dissemination from the primary tumor site to distant loci, this approach provides evidence for the essential role of MET signaling in pulmonary tissue colonization. The lower number of malignant lesions found in mice injected with MET^−/−^ A549 cells compared to those that can fully exploit MET signaling could be due to an impairment of cell proliferation. This does not seem to be the case, as the in vitro growth rate of MET^−/−^ A549 cells was very similar to that of their parental counterparts expressing a functional MET receptor. On the contrary, MET^−/−^ cells cannot take advantage of HGF-elicited MET signaling to promote anchorage-independent growth, survival and invasion, while wild-type A549 cells do. Thus, our in vitro and in vivo data strongly suggest that the fundamental role played by MET during the metastatic process should not be linked to a proliferation boost but should be mostly related to other indispensable functions that up-grade transformed cells to a more malignant phenotype related to the capability to leave the site of the primary lesion, spread, and take root at a secondary locus. These findings are in line with the results obtained by Xu et al., showing that pharmacological inhibition of the HGF-MET axis does not impact cell proliferation while profoundly modulates cancer cell ability of invasiveness, survival, stemness and EMT [19].

The experimental model based on the orthotopic implant of MET^−/−^ Capan-I cells better recapitulates the full metastatic process, as in this case, the nodules established far from the injection site result from the invasion of the original site, intravasation, extravasation, colonization, and outgrowth at the secondary site. We observed that MET^−/−^ Capan-I tumors displayed an impaired ability to expand at the primary site. It is conceivable to hypothesize that the implant of foreign cells into an organ requires the same set of molecular support, including a fully active MET signal, needed during the distant colonization steps. In addition, histological analysis showed a very low number of mitotic events in MET^−/−^ tumors. Thus, even if the in vitro proliferation rates of MET^−/−^ and MET wild-type Capan-I cells were similar and not modulated by HGF, it seems that MET has a role during in vivo cell duplication in this model. The discrepancy between in vitro and in vivo results could be due to the fundamental involvement of the stroma compartment in pancreatic carcinoma [38,39]. In this context, MET is one of the actors playing a crucial role in sustaining the tumor/stroma interplay [40]. We previously showed that, following MET activation, Capan-I cells upregulate Tenascin C secretion and that this molecule promotes the stroma rewiring needed to fully support both primary tumor growth and cancer cell dissemination [35]. Thus, considering this activity, MET plays extrinsic seed features (see above). The lack of MET signaling could impact the ‘non-cell-autonomous’ functions required during pancreatic cancer establishment and progression, a role that could not be assessed by our in vitro analyses.

Cancer cell dissemination is responsible for the vast majority of cancer-related deaths [41]. Such unfavorable outcomes are due to the extremely difficult management of late-stage cancers when only a few treatments show any efficacy. In the vast majority of cases, the same drugs approved for the treatment of the primary disease are applied in the context of the metastatic disease, regardless of the main issue of heterogeneity that characterizes tumor progression and of the fact that dissemination to distant sites is a process that goes far beyond the control of cell proliferation. According to the concept of precision medicine, a targeted drug is efficacious in blocking tumor expansion exclusively if it hits a gene that drives malignancy [42]. By following the same reasoning, a potentially successful approach to hinder metastasis could be to target molecules playing a crucial role during the process of cell dissemination independently of the genetic lesions responsible for transformation. This strategy potentially represents an effective tool against a variety of tumor types, with the advantage of being widely applicable. Currently, several MET-specific TKIs are approved for clinical application [43,44] and some MET-targeting antibodies are under observation in late-stage clinical trials [45]. These molecules could represent valuable tools to be used in combination with drugs targeting the gene identified as the driver of transformation, either to prevent and/or to treat metastasis. Moreover, inhibition of the HGF/MET axis could be combined with chemotherapeutics agents, as proposed in a preclinical study for pancreatic cancer treatment [19]. This setting could not only reduce cancer dissemination but, by downsizing both the primary tumor and the micrometastases, has been proposed as neoadjuvant treatment, potentially improving the outcome of surgical resection.

## 5. Conclusions

Overall, these results directly define the HGF/MET axis as a driving force of the cell-autonomous metastatic process and suggest that it enhances the malignant phenotype by boosting pro-invasive cues, even when it is not the driver of cancer growth. In this context, our study supports the use of MET inhibitors to prevent and/or treat metastatic disease independently of dominant oncogenic signaling. The future exploration of MET inhibition in combination with targeted agents or drugs suitable to block cell proliferation in preclinical models other than those described here will generate valuable knowledge to support its application in the clinic for the treatment of a larger spectrum of cancers.

## Data Availability

The data presented in this study are available in this article (and Supplementary Materials).

## Supplementary Materials

The following supporting information can be downloaded at: https://www.mdpi.com/article/10.3390/cancers15102742/s1, Figure S1: Generation of a MET ^−/−^ human lung carcinoma cell line; Figure S2: Proliferation analysis of wild-type and MET^−/−^ A549 cells; Figure S3: In vitro analysis of HGF-driven biological responses in wild-type and MET^−/−^ A549 cells; Figure S4: In vitro analysis of HGF-driven invasion properties of MET^−/−^ A549 cells in which MET expression has been resumed by lentiviral vector transduction; Figure S5: In vitro analysis of anoikis in wild-type and MET^−/−^ A549 cells; Figure S6: Analysis of luciferase expression by wild-type and MET^−/−^ A549 cells upon transduction of lentiviral vectors expressing Luciferase; Figure S7: Lung colonization assay with wild-type and MET^−/−^ A549 cells; Figure S8: Generation of a MET^−/−^ human PDAC lung carcinoma cell line; Figure S9: Analysis of proliferation of wild-type and MET^−/−^ Capan-I cells; Figure S10: In vitro analysis of HGF-driven biological responses in wild-type and MET^−/−^ Capan-I cells; Figure S11: Analysis of luciferase expression by wild-type and MET^−/−^ Capan-I cells upon transduction of lentiviral vectors expressing Luciferase; Figure S12: In vivo analysis of wild-type and MET^−/−^ Capan-I tumors and metastasis; Figure S13: Histological analysis of wild-type and MET^−/−^ Capan-I tumors. File S1: Western Blot raw data and quantification of the bands (overexposed images are also available).
