# Supplementary material for: Genetic Ablation of the MET Oncogene Defines a Crucial Role of the HGF/MET Axis in Cell-Autonomous Functions Driving Tumor Dissemination
Source: Cancers (Basel). 2023 May 13;15(10):2742. doi: 10.3390/cancers15102742 (PMC10216813; doi:10.3390/cancers15102742)
Supplement: Supplementary file 1 [file cancers-15-02742-s001.zip › Suppl. Material File S1_WB-rawdata-quantification_20230512.pdf]

Western blot Figure 1B

MW 1 2

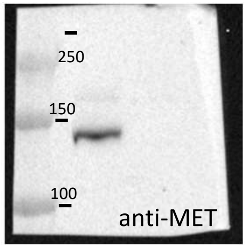

MW 1 2

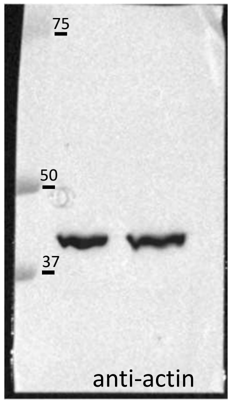

wt MET$^{-/-}$

p145 MET

p40 actin

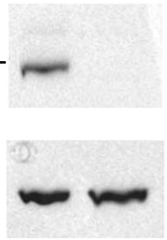

Figure 1 b	actin	MET	MET/Actin
wt	66.368.200	64.885.969	0,978
MET$^{-/-}$	146.231.871	2.931.681	0,020

MW: Molecular Weight (Kda)

1: A549 wild type

2: A549 MET$^{-/-}$

Western blot Figure 1C

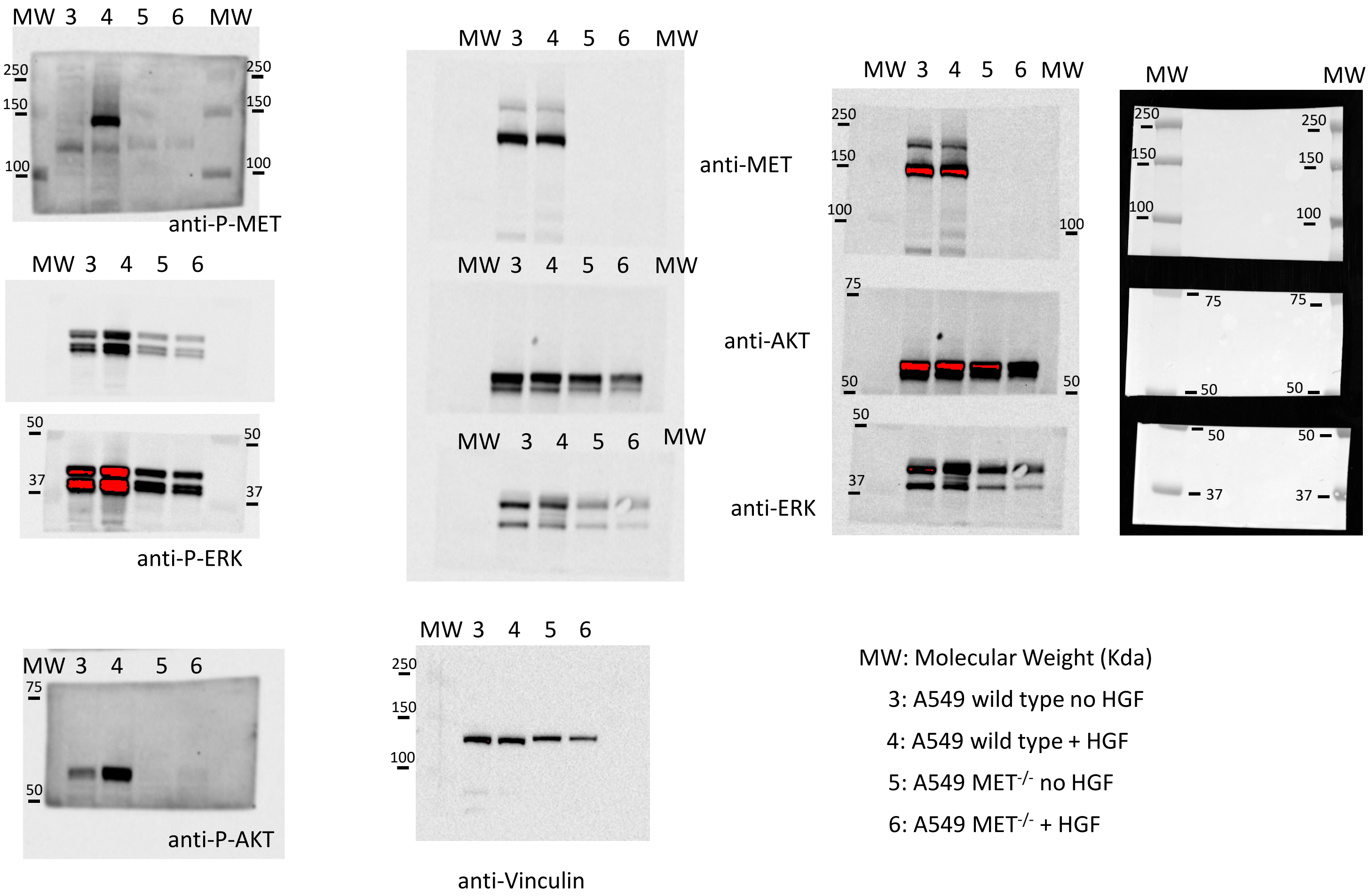

MW: Molecular Weight (Kda)

3: A549 wild type no HGF

4: A549 wild type + HGF

5: A549 MET$^{-/-}$ no HGF

6: A549 MET$^{-/-}$ + HGF

Western blot Figure 1C

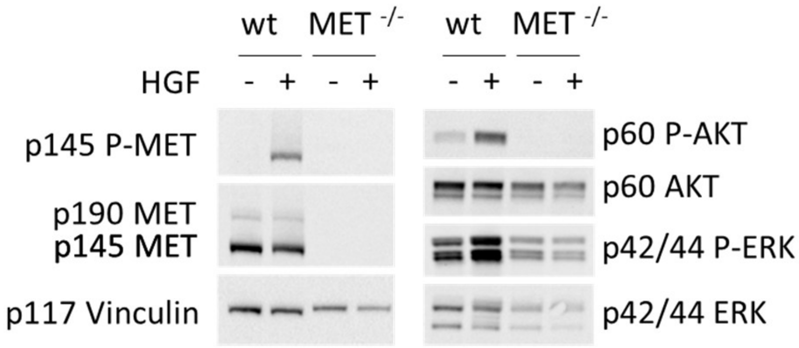

Figure 1 c	vinculin	pMET	MET	pERK	ERK	pAKT	AKT
wt	30.258.530	3.127.196	39.208.510	24.128.731	31.406.924	10.896.196	34.977.731
wt + HGF	24.899.560	36.842.773	31.735.167	38.627.711	31.846.510	40.961.652	32.034.317
$MET^{-/-}$	17.570.267	912.510	0	12.376.518	14.664.095	1.165.660	23.578.246
$MET^{-/-}$ + HGF	9.853.024	377.163	0	7.881.569	8.501.217	1.165.660	15.867.811

Figure 1 c	pMET/Vinc	MET/Vinc	pERK/Vinc	ERK/Vinc	pAKT/Vinc	AKT/Vinc
wt	0,103	1,296	0,797	1,038	0,360	1,156
wt + HGF	1,480	1,275	1,551	1,279	1,645	1,287
$MET^{-/-}$	0,052	0,000	0,704	0,835	0,066	1,342
$MET^{-/-}$ + HGF	0,038	0,000	0,800	0,863	0,118	1,610

Figure 1 c	pMET/MET	pERK/ERK	pAKT/AKT
wt	0,080	0,768	0,312
wt + HGF	1,161	1,213	1,279
$MET^{-/-}$	#DIV/0!	0,844	0,049
$MET^{-/-}$ + HGF	#DIV/0!	0,927	0,073

Western Blot Figure 5B

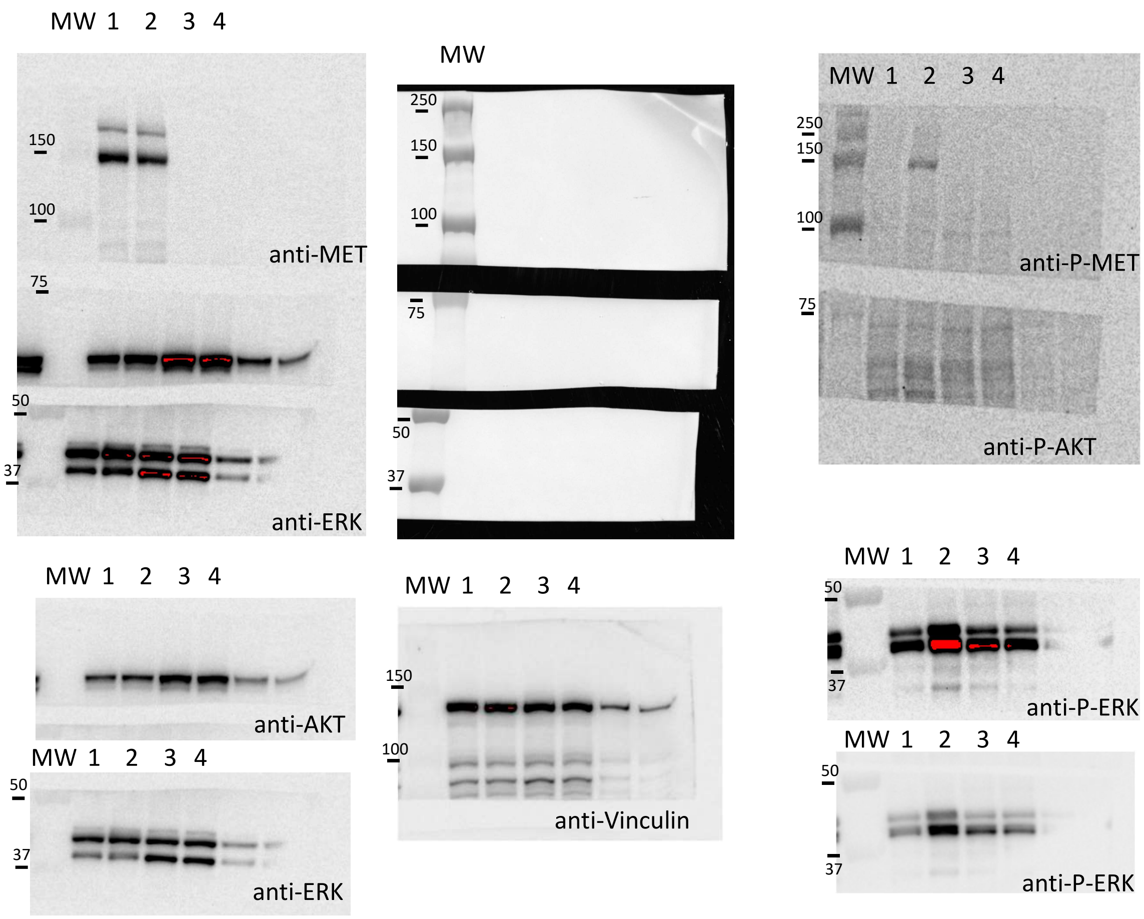

MW: Molecular Weight (Kda)

1: Capan-I wild type no HGF

2: Capan-I wild type + HGF

3: Capan-I $MET^{-/-}$ no HGF

4: Capan-I $MET^{-/-}$ + HGF

Western Blot Figure 5B

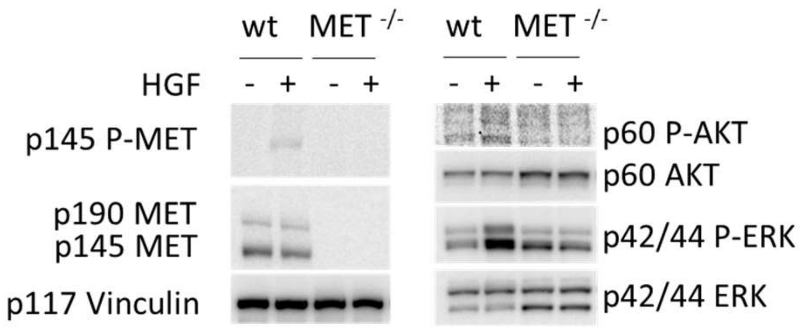

Figure 4 b	Vinculin	pMET	MET	pAKT	AKT	pERK	ERK
wt	40.803.468	5.197.719	38.570.066	9.973.104	7.607.640	14.087.539	9.530.276
wt + HGF	42.103.317	17.134.468	33.836.894	15.007.640	9.454.154	40.519.631	11.564.104
MET$^{-/-}$	41.045.660	0	1.403.589	9.325.640	16.568.225	22.517.974	15.031.468
MET$^{-/-}$ + HGF	87.629.453	1	1.403.589	7.666.933	14.305.569	17.510.196	14.214.518

Figure 4 b	pMET/Vinc	MET/Vinc	pAKT/Vinc	AKT/Vinc	pERK/Vinc	ERK/Vinc
wt	0,127	0,945	0,244	0,186	0,345	0,234
wt + HGF	0,407	0,804	0,356	0,225	0,962	0,275
MET$^{-/-}$	0,000	0,034	0,227	0,404	0,549	0,366
MET$^{-/-}$ + HGF	0,000	0,016	0,087	0,163	0,200	0,162

Figure 4 b	pMET/MET	pAKT/AKT	pERK/ERK
wt	0,135	1,311	1,478
wt + HGF	0,506	1,587	3,504
MET$^{-/-}$	0,000	0,563	1,498
MET$^{-/-}$ + HGF	0,000	0,536	1,232

Western Blot Supplementary Figure S8B

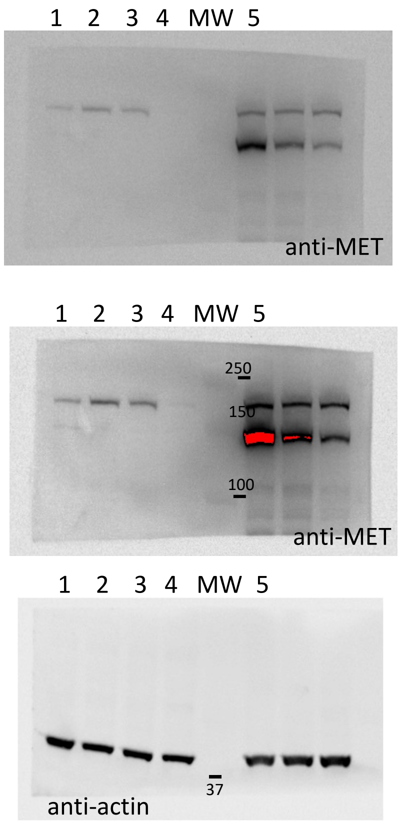

MW: Molecular Weight (Kda)

1: Capan-I clone 5

2: Capan-I clone 8

3: Capan-I clone 11

4: Capan-I clone 13

5: Capan-I wild type

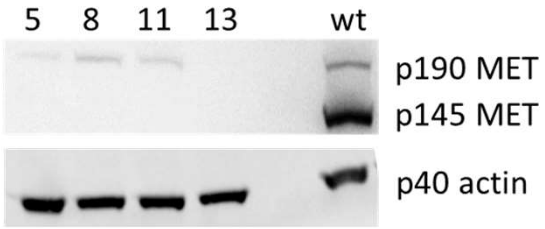

Suppl. Fig. 3B	MET	Actin	MET/Actin
Clone 5		31.870.539	0
Clone 8		29.446.125	0
Clone 11		29.178.317	0
Clone 13		29.024.439	0
WT	31.702.681	68.976.127	0,460
