# Supplementary material for: Genetic Ablation of the MET Oncogene Defines a Crucial Role of the HGF/MET Axis in Cell-Autonomous Functions Driving Tumor Dissemination
Source: Cancers (Basel). 2023 May 13;15(10):2742. doi: 10.3390/cancers15102742 (PMC10216813; doi:10.3390/cancers15102742)
Supplement: Supplementary file 1 [file cancers-15-02742-s001.zip › Supplementary Figures.pdf]

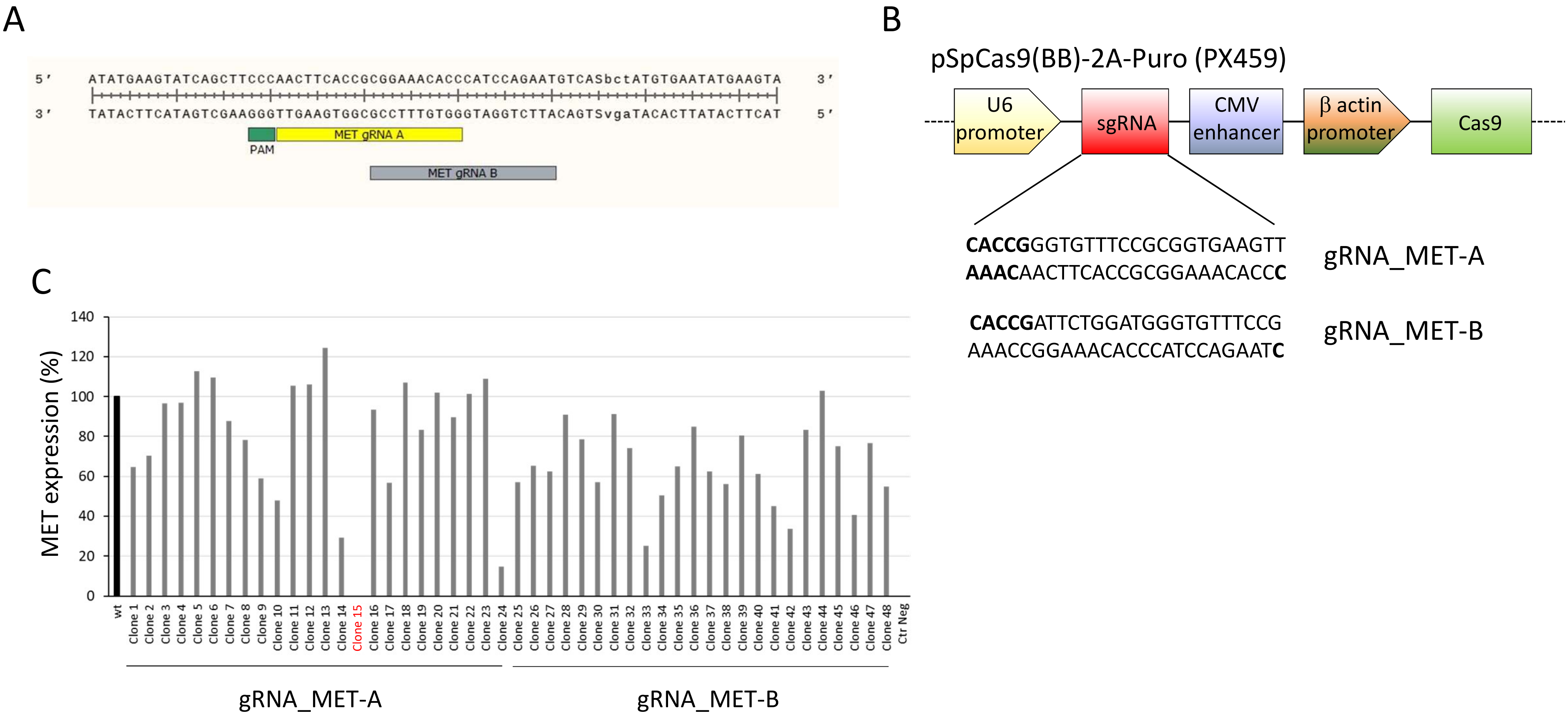

Figure S1 - Generation of a MET$^{-/-}$ human lung carcinoma cell line. **A.** Schematic representation of the two RNA guide location on the exon 2 of the MET gene. **B.** Schematic representation of the main features of the plasmid used for CRISPR-Cas9 directed gene editing of A549 human lung adenocarcinoma cells. The nucleotide sequence of the guide RNAs targeting the second exon of MET gene is reported. **C.** MET protein expression in each single clone measured by ELISA. The graph reports MET expression as the % value of the level measured in wild-type A549 cells. The MET negative clone is highlighted in red.

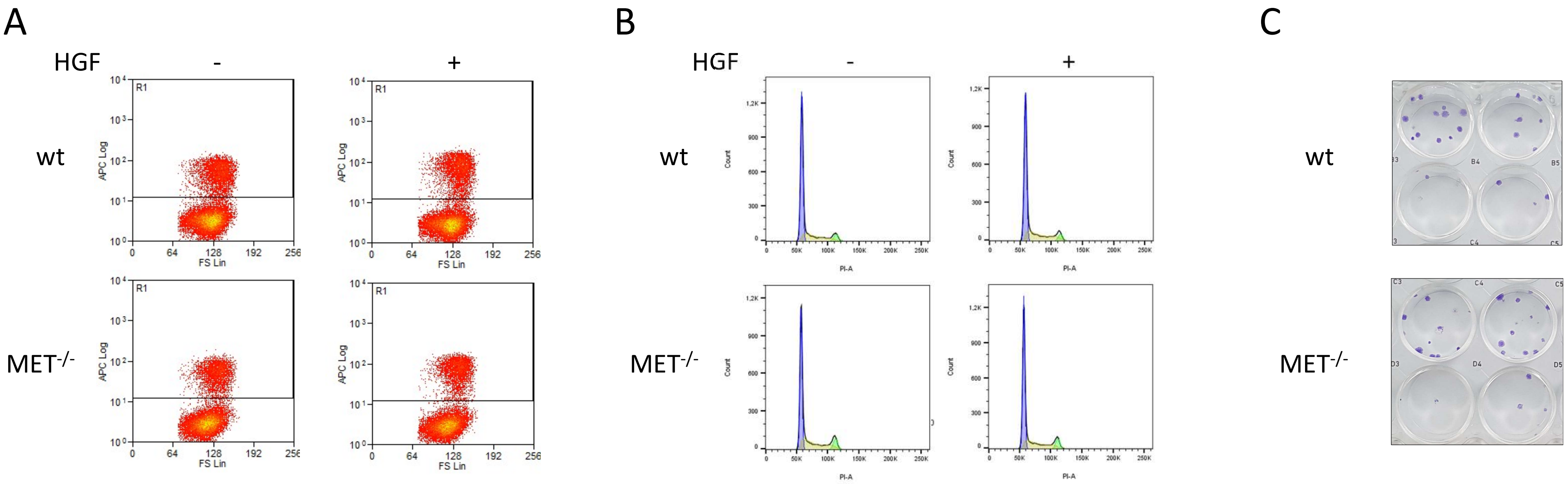

Figure S2 – Proliferation analysis of wild-type (wt) and MET$^{-/-}$ A549 cells. **A.** Cytofluorimeter analysis of EdU incorporation after 48 hrs of culture in medium without serum, in the presence or the absence of HGF (25ng/ml). Pictures are representative images of the analysis. **B.** Cell cycle profile after 48 hrs of culture in medium with 2% serum, in the presence or the absence of HGF (25ng/ml). Pictures are representative images of the analysis. **C.** Clonogenic potential of wild-type and MET$^{-/-}$ A549 cells. Representative images of clones obtained upon 10 days of culture in 10% FCS medium, seeding 20 or 4 cells/well (top or bottom wells, respectively). Data reported in the figure are representative of two experiments.

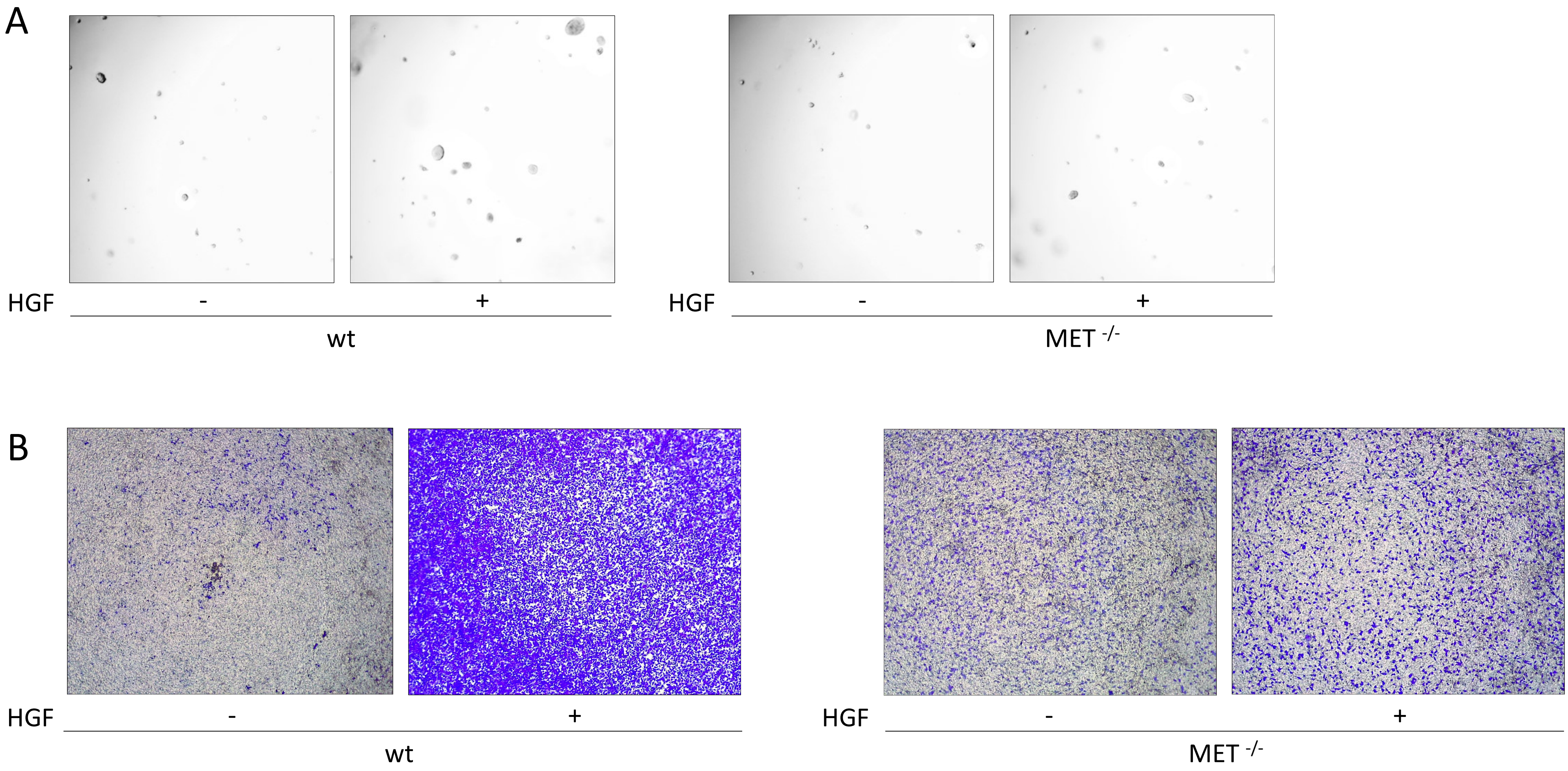

Figure S3 - In vitro analysis of HGF-driven biological responses in wild-type and $MET^{-/-}$ A549 cells. **A.** Analysis of anchorage-independent growth by soft agar assay with wild-type (wt) or $MET^{-/-}$ A549 cells grown for 14 days in the presence or in the absence of HGF (25 ng/ml). One representative image per treatment is shown. **B.** Analysis of cell invasion by transwell assay with wild-type (wt) or $MET^{-/-}$ A549 cells migrated through a Matrigel layer during 24 hrs of culture in the presence or in the absence of HGF (25 ng/ml). One representative image per treatment is shown. Data reported in the figure are representative of at least two experiments.

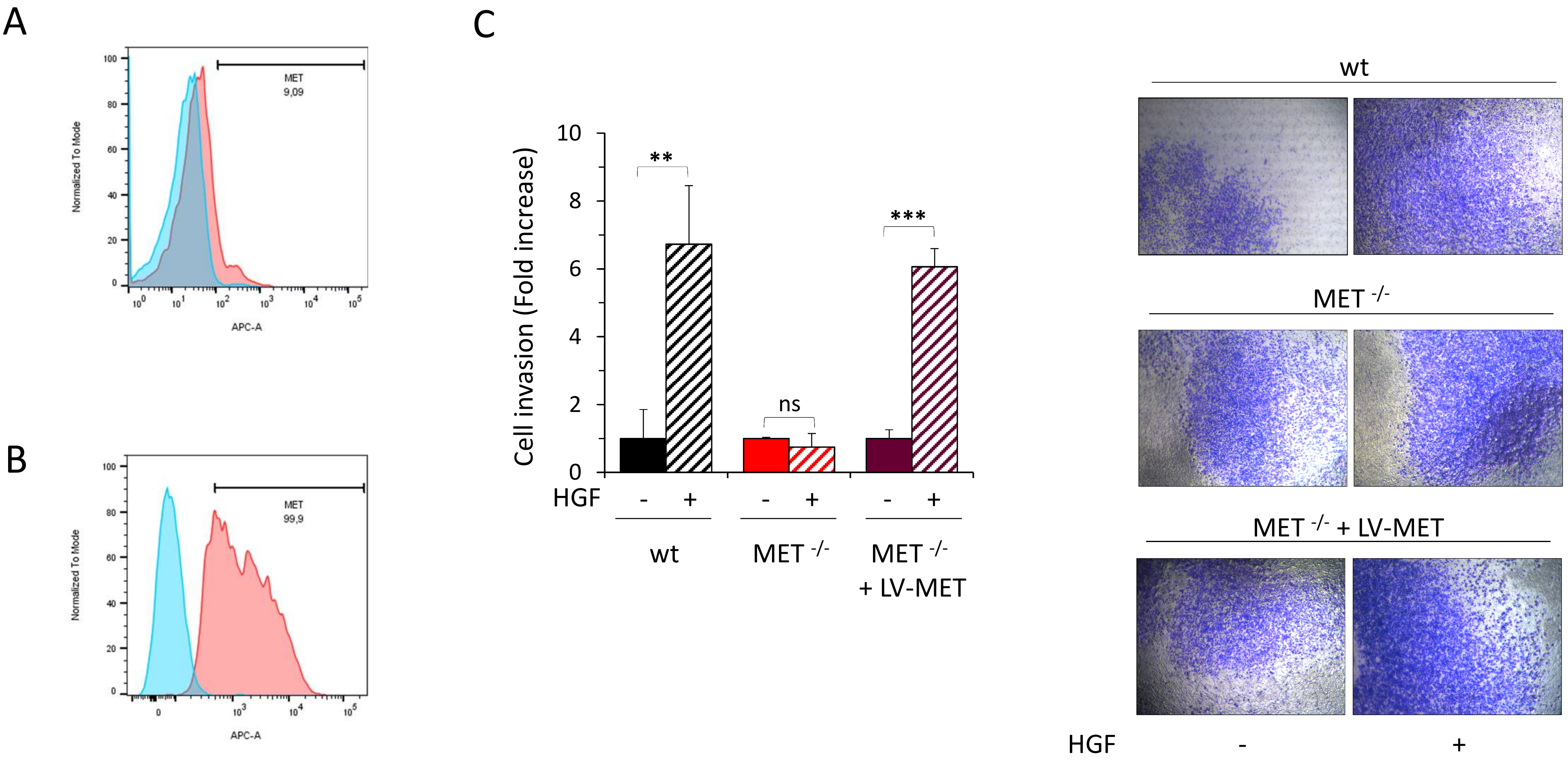

Figure S4 - In vitro analysis of HGF-driven invasion properties of $MET^{-/-}$ A549 cells in which MET expression has been resumed by lentiviral vector transduction. **A.** MET expression by cytofluorimeter analysis in $MET^{-/-}$ A549 cells transduced with lentiviral vector particles carrying MET c-DNA. Light blue: original $MET^{-/-}$ A549 cell population; red: transduced cell population. The percentage of MET positive cells is indicated in the plot. **B.** MET expression by cytofluorimeter analysis in $MET^{-/-}$ A549 cells transduced with lentiviral vector particles carrying MET c-DNA upon sorting with anti-MET antibodies. Light blue: isotype control; red: sorted cell population. The percentage of MET positive cells is indicated in the plot. **C.** Analysis of cell invasion by transwell assay with wild-type (wt) or $MET^{-/-}$ A549 cells migrated through a Matrigel layer during 24 hrs of culture in the presence of HGF (25 ng/ml, in both chambers) and serum (5% FCS in the lower chamber and 1% FCS in the upper chamber). Graph on the left reports fold increase of migrated cells, with respect to the untreated counterpart. Each point is the mean of triplicates; bars represent SD. ***, $p \leq 0.001$; **, $p \leq 0.01$; ns: not significant. On the right, one representative image per treatment is shown. Data reported in the figure are representative of two experiments.

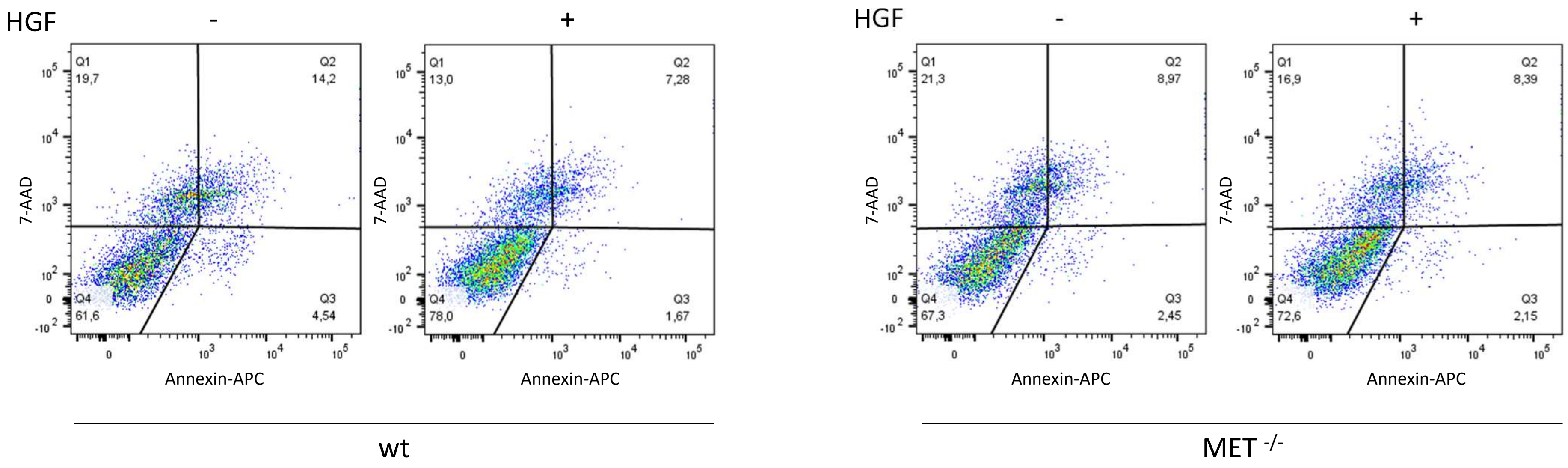

Figure S5 - In vitro analysis of anoikis in wild-type and MET$^{-/-}$ A549 cells. **A.** Analysis of annexin positive wild-type (wt) or MET$^{-/-}$ A549 cells grown for 48 hours in the absence of matrix adhesion, with or without HGF (25 ng/ml). One representative image per treatment is shown. Numbers in the plots represent the percentage of events in each quadrant. Data reported in the figure are representative of two experiments.

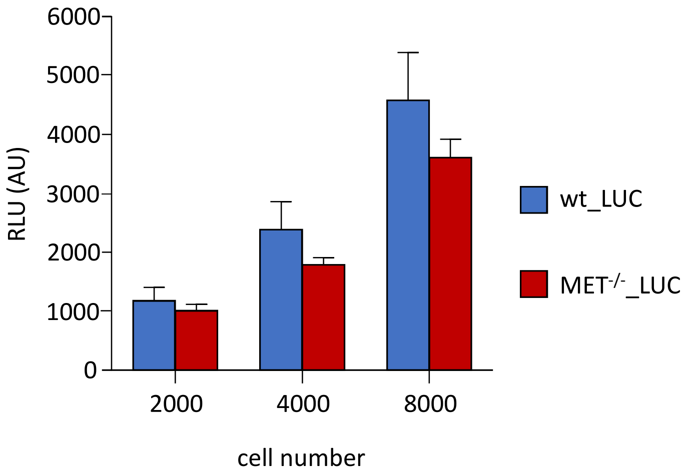

Figure S6 - Analysis of luciferase expression by wild-type and MET$^{-/-}$ A549 cells upon transduction of lentiviral vectors expressing Luciferase (LUC). Graph reports the relative light units (RLU) measured in different amounts of cells. AU: arbitrary unit.

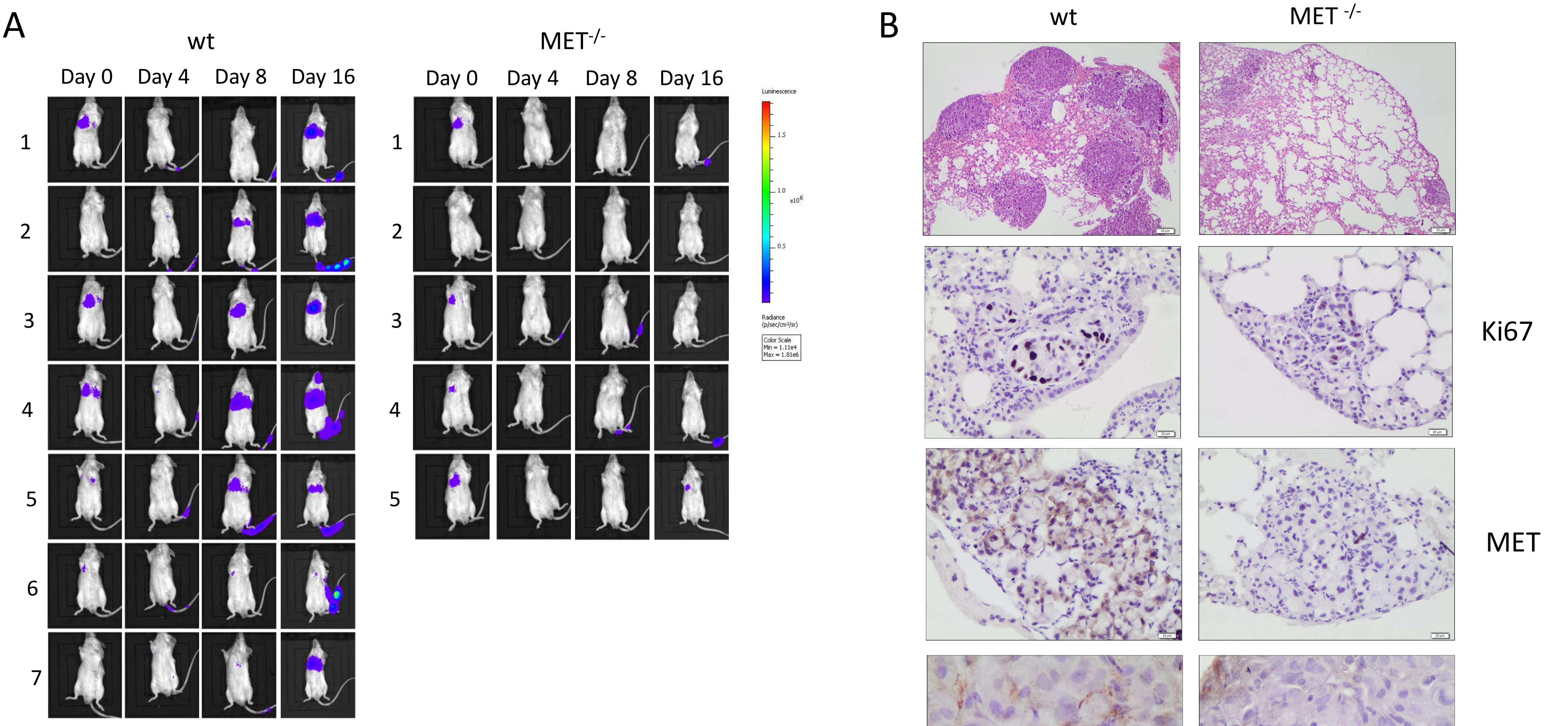

Figure S7 - Lung colonization assay with wild-type and MET$^{-/-}$ A549 cells. Luciferase-expressing wild-type and MET$^{-/-}$ A549 cells were injected into the tail vein of hHGF-KI mice. **A.** IVIS images of each mouse performed 4h post-injection (day 0) and then after 4-8-16 days. **B.** Histological evaluation and immunohistochemistry analysis by anti-Ki67, anti-MET, and anti-PhosphoMET antibodies of lungs excised five weeks after cell injection.

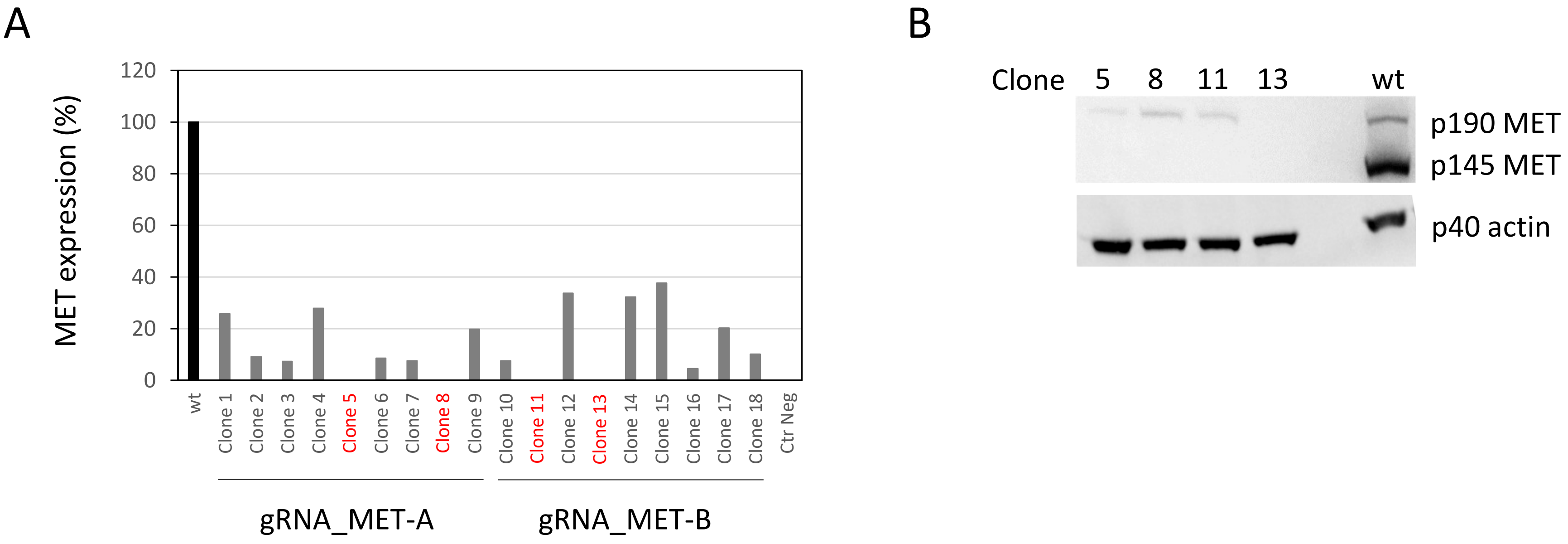

Figure S8 - Generation of a MET$^{-/-}$ human PDAC lung carcinoma cell line. **A.** MET protein expression measured by ELISA in each single clone obtained after puromycin selection of Capan-I cells transfected with the plasmids carrying gRNA_MET-A or gRNA_MET-B sequences. The graph reports MET expression as the % value of the level measured in wild-type Capan-I cells. MET negative clones are highlighted in red. **B.** Immunoblotting analysis of MET expression in lysates from putative MET$^{-/-}$ Capan-I clones. As loading control actin analysis is shown. p190 MET: precursor form of the MET receptor; p145 MET: MET receptor β chain; p40 actin: Actin.

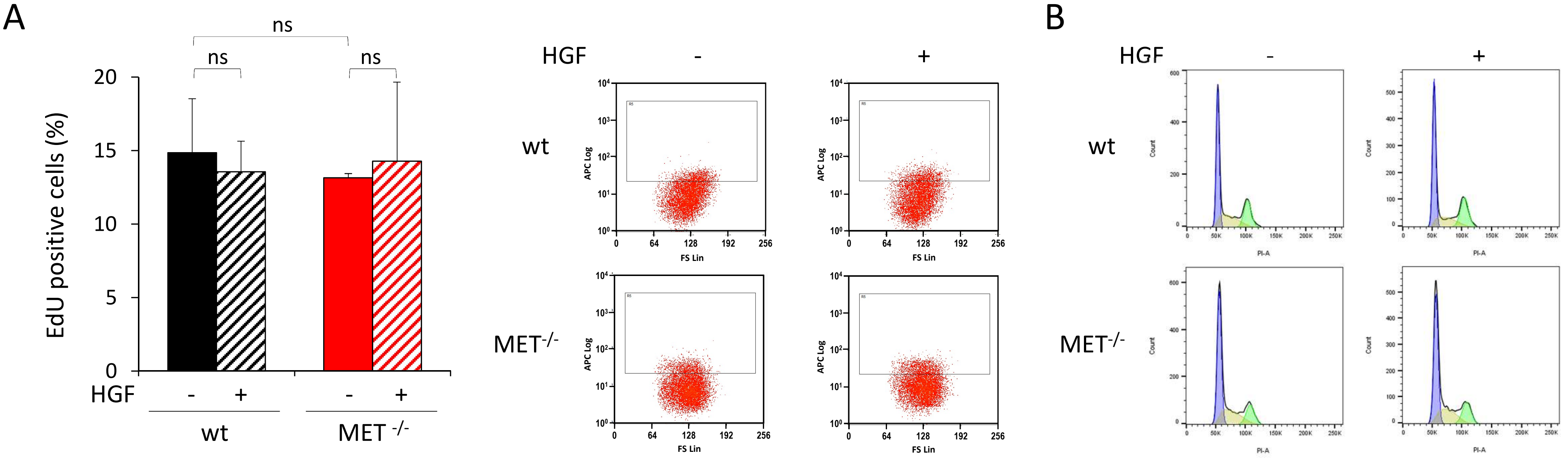

Figure S9 – Analysis of proliferation of wild-type and MET$^{-/-}$ Capan-I cells. **A.** Cytofluorimeter analysis of EdU incorporation after 48 hrs of culture in medium with 2% serum, in the presence or the absence of HGF (25ng/ml). Graph on the left reports the percentage of EdU positive cells. Each point is the mean of duplicate values; bars represent SD; ns: not significant. On the right, pictures representative of the analysis. **B.** Cell cycle profile after 48 hrs of culture in medium with 2% serum, in the presence or the absence of HGF (25ng/ml). Samples were in triplicate. Data reported in the figure are representative of two experiments.

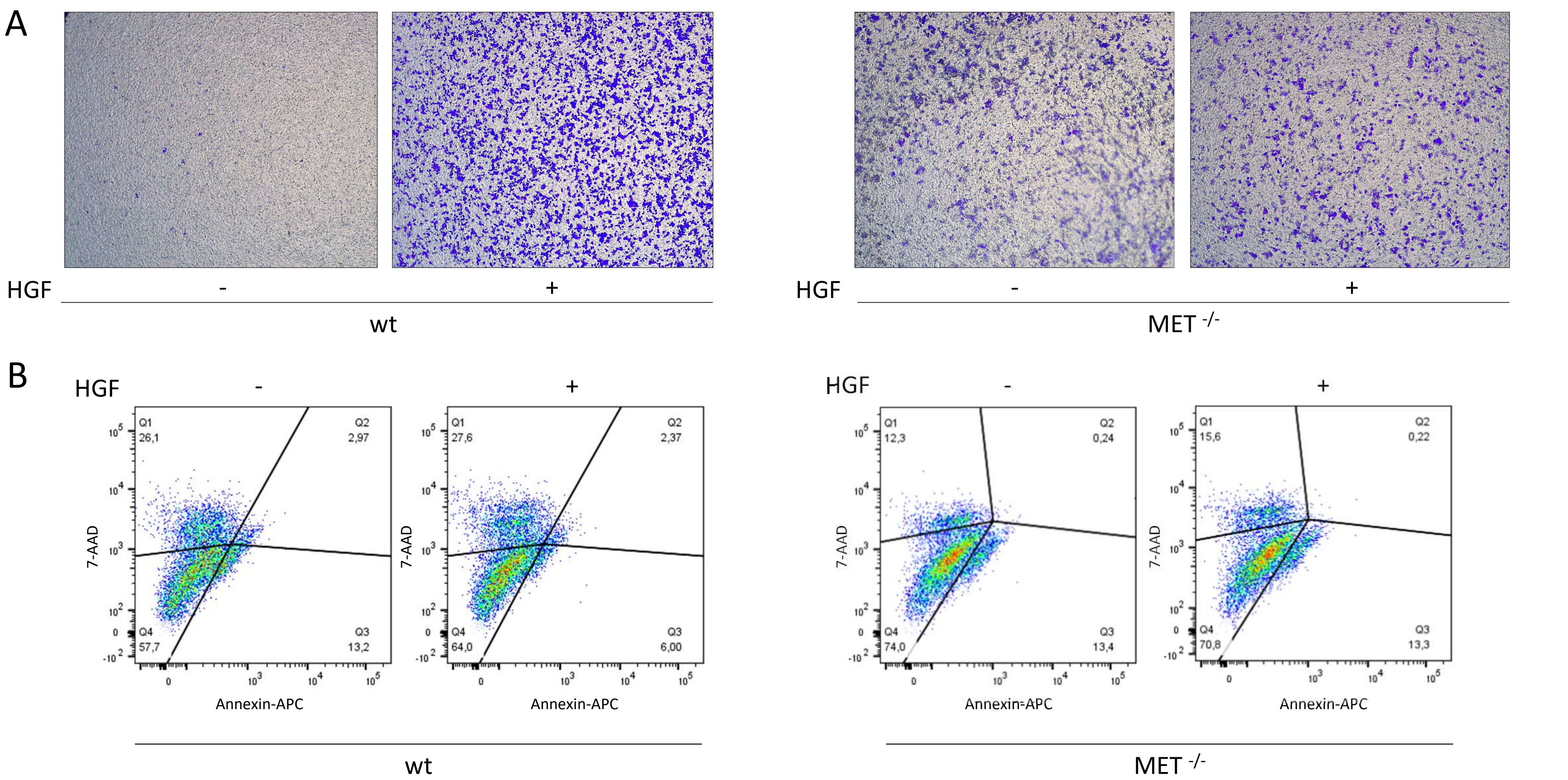

Figure S10 – In vitro analysis of HGF-driven biological responses in wild-type and $MET^{-/-}$ Capan-I cells. **A.** Cell invasion by transwell assay with wild-type (wt) or $MET^{-/-}$ Capan-I cells migrated through a Matrigel layer during 24 hrs of culture with or without HGF (25 ng/ml). One representative image per treatment is shown. **B.** Analysis of annexin positive wild-type (wt) or $MET^{-/-}$ Capan-I cells grown for 48 hours in the absence of matrix adhesion with or without HGF (25 ng/ml). One representative image per treatment is shown. Numbers in the plots represent the percentage of events in each quadrant. Data reported in the figure are representative of at least two experiments.

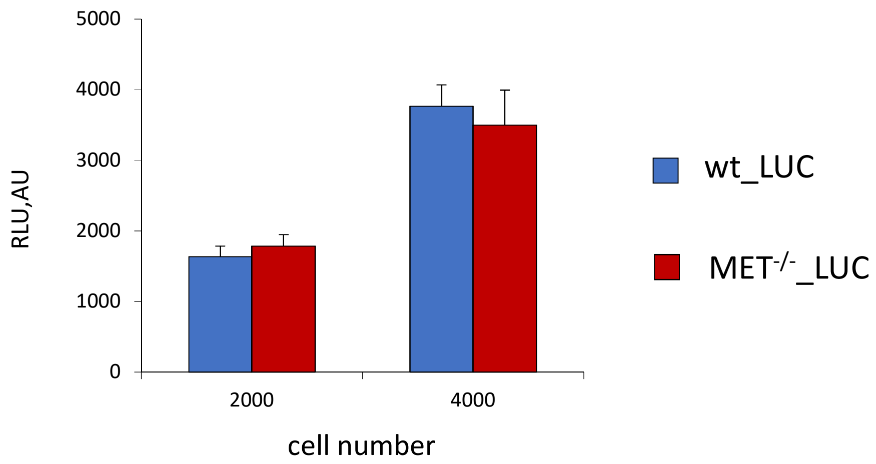

Figure S11 - Analysis of luciferase expression by wild-type (wt) and $MET^{-/-}$ Capan-I cells upon transduction of lentiviral vectors expressing Luciferase (LUC). Graph reports the relative light units (RLU) measured in different amounts of cells. AU: arbitrary unit.

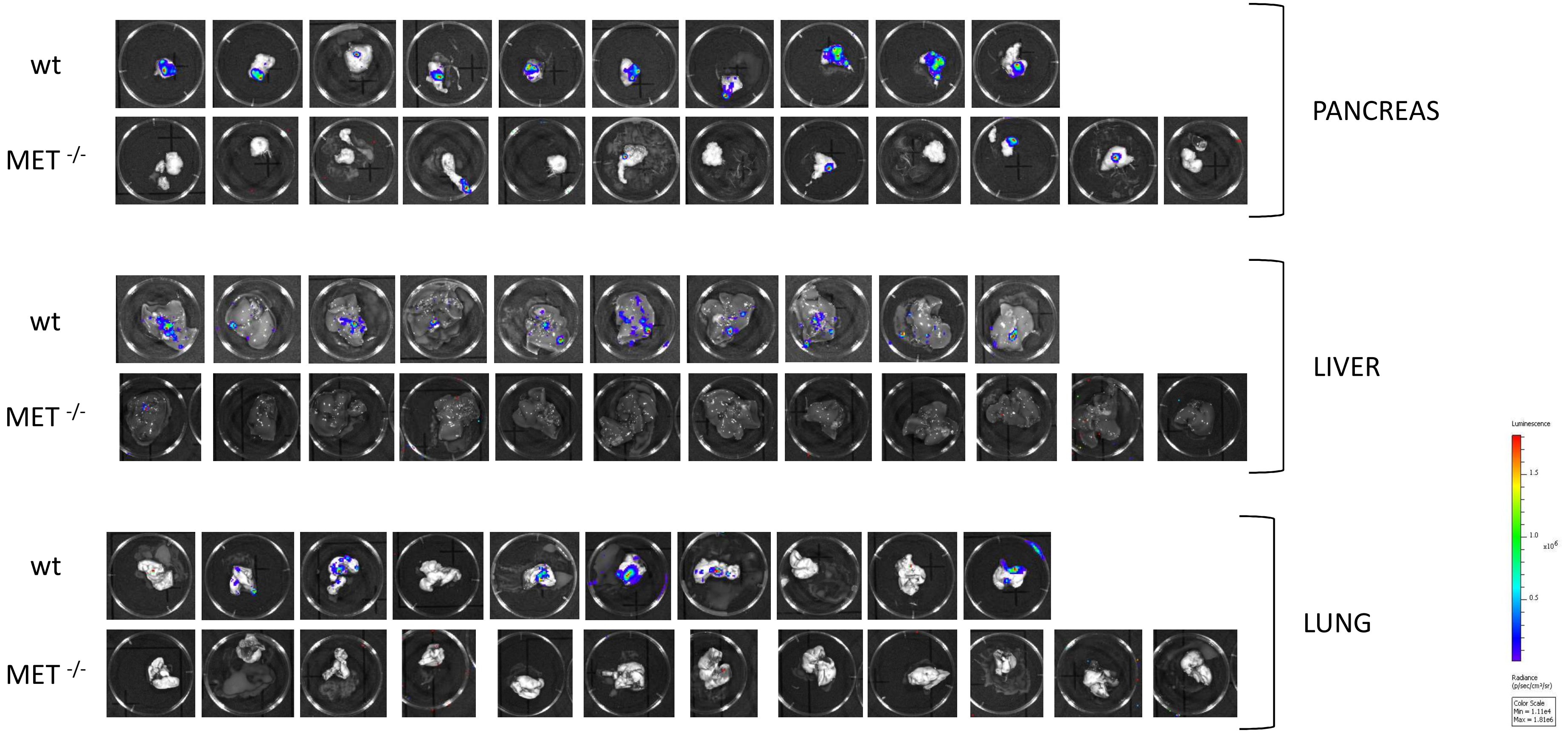

Figure S12 - In vivo analysis of wild-type and MET$^{-/-}$ Capan-I tumors and metastasis. Luciferase-expressing wild-type and MET $^{-/-}$ Capan-I cells were injected into the pancreas of hHGF-KI mice. IVIS images of isolated organs (pancreas, livers, and lungs) excised from mice at day 35.

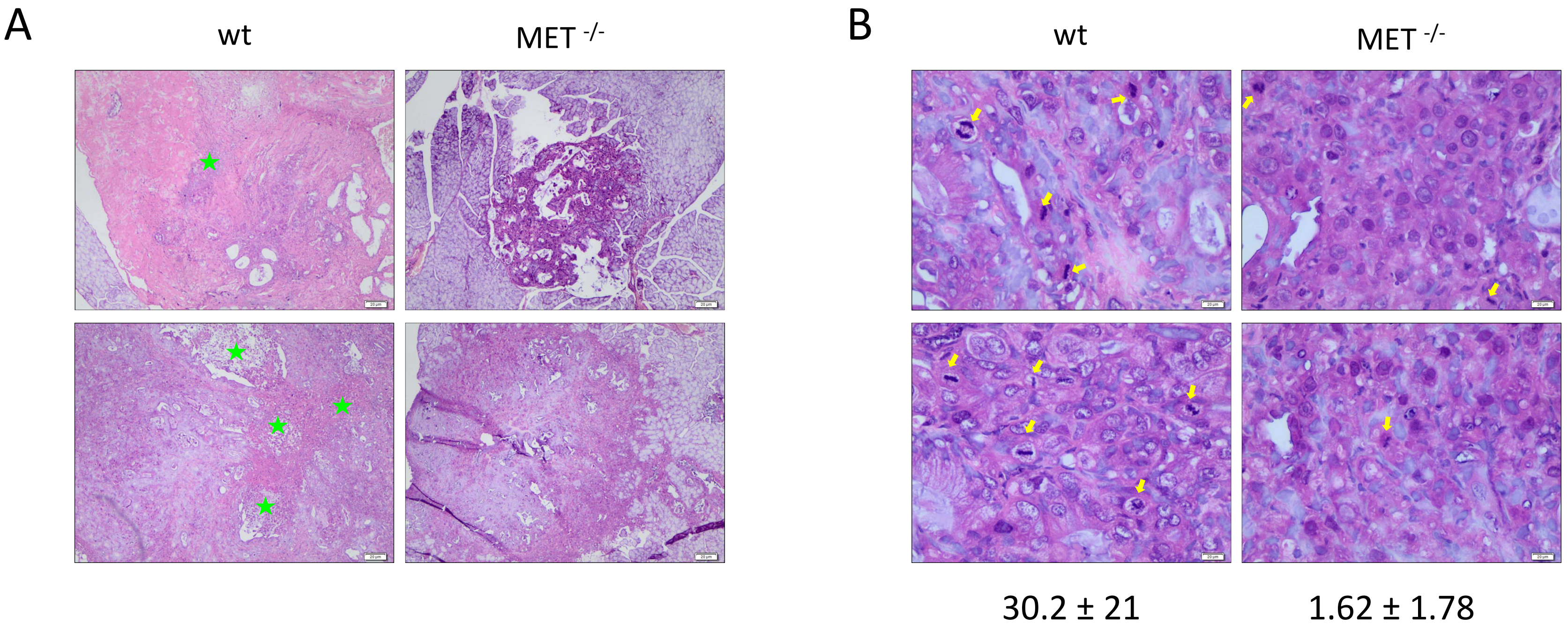

Figure S13 – Histological analysis of wild-type and MET$^{-/-}$ Capan-I tumors. **A.** Representative images of tumors in pancreas excised from mice 35 days after the injection of wild-type or MET$^{-/-}$ Capan-I cells. Green stars indicates necrotic areas.) **B.** Representative images of tumors at higher magnification. Yellow arrows indicates duplicating cells. Numbers represents the average number of mitotic cells counted in 10 High Power Fields +/- standard deviation.
